# Endocardial identity is established during early somitogenesis by Bmp signalling acting upstream of *npas4l* and *etv2*

**DOI:** 10.1242/dev.190421

**Published:** 2022-05-09

**Authors:** Samuel J. Capon, Veronica Uribe, Nicole Dominado, Ophelia Ehrlich, Kelly A. Smith

**Affiliations:** 1 Division of Genomics of Development and Disease, Institute for Molecular Bioscience, The University of Queensland, Brisbane, Queensland 4072, Australia; 2 Department of Anatomy & Physiology, The University of Melbourne, Melbourne, Victoria 3010, Australia

**Keywords:** Endocardium, *etv2/etsrp*, *npas4l*/*cloche*, Bmp signalling, Zebrafish, Cardiac

## Abstract

The endocardium plays important roles in the development and function of the vertebrate heart; however, few molecular markers of this tissue have been identified and little is known about what regulates its differentiation. Here, we describe the *Gt(SAGFF27C); Tg(4xUAS:egfp)* line as a marker of endocardial development in zebrafish. Transcriptomic comparison between endocardium and pan-endothelium confirms molecular distinction between these populations and time-course analysis suggests differentiation as early as eight somites. To investigate what regulates endocardial identity, we employed *npas4l*, *etv2* and *scl* loss-of-function models. Endocardial expression is lost in *npas4l* mutants, significantly reduced in *etv2* mutants and only modestly affected upon *scl* loss-of-function. Bmp signalling was also examined: overactivation of Bmp signalling increased endocardial expression, whereas Bmp inhibition decreased expression. Finally, epistasis experiments showed that overactivation of Bmp signalling was incapable of restoring endocardial expression in *etv2* mutants. By contrast, overexpression of either *npas4l* or *etv2* was sufficient to rescue endocardial expression upon Bmp inhibition. Together, these results describe the differentiation of the endocardium, distinct from vasculature, and place *npas4l* and *etv2* downstream of Bmp signalling in regulating its differentiation.

## Abstract (precis)

**Summary:** A zebrafish transgenic reporter of the endocardium is identified, permitting transcriptomic analysis and identification of new endocardial markers. Epistasis experiments demonstrate *npas4l* and *etv2* act downstream of Bmp signalling to regulate endocardial differentiation.

## INTRODUCTION

During early embryonic development, the heart comprises two major tissues: the myocardium and endocardium. To date, much research concerning heart development has focused on the myocardium, the beating muscle of the heart, whereas the endocardium has received relatively little attention. The endocardium is a specialised subset of endothelium that forms the inner lining of the heart. It plays important roles in the development of the trabecular myocardium, contributes to the coronary vasculature, septa and cardiac cushions, and connects the heart with the adjacent vascular endothelial network (Harris and Black, 2010; Kisanuki et al., 2001; Markwald et al., 1977, 1975; Meyer and Birchmeier, 1995; Red-Horse et al., 2010; Stankunas et al., 2008; Tian et al., 2017). Despite these important and unique roles, the developmental cues that distinguish the endocardium from the vascular endothelium remain to be determined.

Molecularly, the earliest specific marker of endocardium is *Nfatc1* (de la Pompa et al., 1998; Wong et al., 2012). The endocardial-specific expression of this marker, combined with the functionally distinct roles of the endocardium, argue that the endocardium is a unique subset of endothelium with its own molecular signature. When and how this distinction is first established, however, remains unclear. In mice, *Nfatc1* expression is already detectable in the medial aspect of the cardiac crescent at embryonic day (E)7.5 (de la Pompa et al., 1998), the earliest recognised structure of the murine heart. In contrast, the zebrafish orthologue of this gene, *nfatc1*, is not reported to initiate expression until 22 hours post fertilisation (hpf) (Wong et al., 2012), corresponding with the formation of the linear heart tube. In zebrafish, endocardial progenitors bud from bilateral, *kdrl^+^/fli1a* (*fli1*)*^+^* populations at the 12-somite (s) stage (15 hpf) and migrate to the midline to form the endocardial core of the cardiac disc (Bussmann et al., 2007; Proulx et al., 2010) before forming the linear heart tube. This observation suggests that the endocardial and vascular endothelial progenitors, which remain in the lateral margins of the anterior lateral plate mesoderm (ALPM), are distinct populations as early as 12 s. However, molecular evidence that corroborates this early functional distinction in zebrafish has yet to be reported.

Separation of lineages at this early stage is not unprecedented. In fact, distinct expression domains for myeloid (*spi1b*, *runx1*, *myb*) (Kalev-Zylinska et al., 2002; Lieschke et al., 2002; Thompson et al., 1998), vascular endothelial (*fli1a*, *kdrl*) (Thompson et al., 1998) and myocardial (*nkx2.5*, *myl7*) progenitors are well described by the 14 s stage (16 hpf) (Chen and Fishman, 1996; Yelon et al., 1999). These fields sit adjacent and mutually exclusive to one another, suggesting they are already specified. Importantly, they also respond differently to genetic cues; for example, the anterior myeloid population is dependent on Bmp signalling, whereas the adjacent vascular field at the same developmental stage is not [as determined by *in situ* for *pu.1* (*spi1b*), *scl* (*tal1*) and *fli1a*] (Hogan et al., 2006). Apparently, location is everything in this context because vascular fate is sensitive to Bmp signalling in the posterior lateral plate mesoderm at the same developmental stage (Liu et al., 2008).

Here, we report the identification and characterisation of a previously reported marker of the zebrafish lymphatic system (Bussmann et al., 2010; Okuda et al., 2012) as a novel marker of early endocardial progenitors. Characterisation of this line identified distinct endocardial expression domains as early as 8 s (13 hpf). Similarly, a re-analysis of *nfatc1* expression found overlapping *nfatc1* expression domains. RNA-sequencing of endocardial and endothelial progenitors identified a distinct transcriptional signature in the endocardium, confirming an early divergence in these tissues. Finally, examination of the signals governing endocardial lineage development identified *npas4l* (*cloche*), *etv2* (*etsrp*) and Bmp signalling as important regulators of endocardial development, and epistasis experiments place Bmp signalling upstream of *npas4l* and *etv2* in the ALPM for induction of endocardial marker expression.

## RESULTS

### The *Gt(SAGFF27C); Tg(4xuas:egfp)* line labels the endocardium during early-to-mid somitogenesis stages

**Fig. 1.**
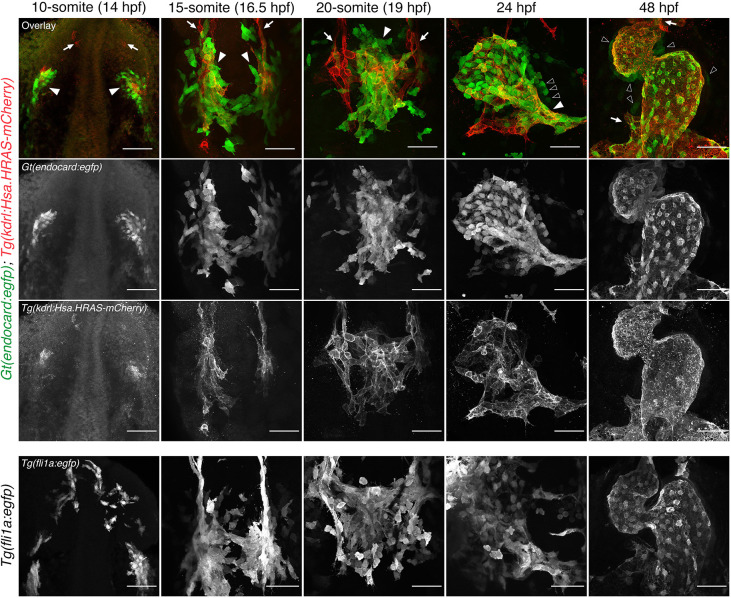
**Time-course analysis of *Gt(endocard:egfp)* fluorescence demonstrates enriched endocardial expression at somitogenesis stages in the zebrafish embryo.** Immunofluorescence staining of *Gt(endocard:egfp); Tg(kdrl:Hsa.HRAS-mCherry)* and *Tg(fli1a:egfp)* embryos from 10 s through to 48 hpf. Anterior to the top. Anterior views at 48 hpf, all other images show dorsal views. White arrowheads label kdrl^+^/endocard:egfp^+^ endocardial cells. White arrows label kdrl^+^/endocard:egfp^−^ vascular endothelial cells. Black arrowheads label presumptive myocardial cells. Scale bars: 50 μm.

The *Gt(SAGFF27C); Tg(4xuas:egfp)* line, originating from a large gene-trap screen (Asakawa et al., 2008), was observed to have enriched expression in the endocardium at 48 hpf, relative to the vascular expression previously described in Bussmann et al. (2010). To investigate this, we performed time-course analysis of the *Gt(SAGFF27C); Tg(4xuas:egfp)* line [henceforth referred to as *Gt(endocard:egfp)*], and compared it with the endothelial transgenic lines *Tg(kdrl:Hsa.HRAS-mCherry)* and *Tg(fli1a:egfp)* (Fig. 1). eGFP expression in the *Gt(endocard:egfp)* line is first visible as bilateral populations in the ALPM at the 10 s stage (14 hpf). It colocalises with *Tg(kdrl:Hsa.HRAS-mCherry)* and localises similar to *Tg(fli1a:egfp)*, confirming that its expression is endothelial, and it is consistent with regions of endocardial progenitors (Fig. 1).

Consistent with previous reports (Bussmann et al., 2007; Proulx et al., 2010), the bilateral populations migrate to the midline by 15 s, whereas vascular progenitors remained at the lateral margins of the ALPM (Fig. 1). *Gt(endocard:egfp)* expression is largely restricted to these migrating endocardial progenitors. By 20 s, endocardial progenitors have fused to form the endocardial core of the cardiac disc (Fig. 1). Although some cells appear to be GFP^+^/mCherry^−^, quantification shows the majority (93%) to be double positive (Fig. S1). Analysis of time-lapse movies shows that this line also likely labels a few myeloid cells at this time, based on their morphology and migratory movements (Movies 1 and 2). At 24 hpf, endocardial expression is observed in the linear heart tube of *Gt(endocard:egfp)* embryos (Fig. 1). By 48 hpf, *Gt(endocard:egfp)* expression has expanded and is also observed in the developing vascular endothelium (Fig. 1). At 24 and 48 hpf, some weak GFP expression can also be observed in the myocardium (Fig. S2). Together, these data suggest that *Gt(endocard:egfp)* is enriched in the endocardium at early-to-mid somitogenesis stages, making it a unique tool for studying endocardial development in a living embryo.

### The endocardium is a molecularly distinct subset of endothelium

**Fig. 2.**
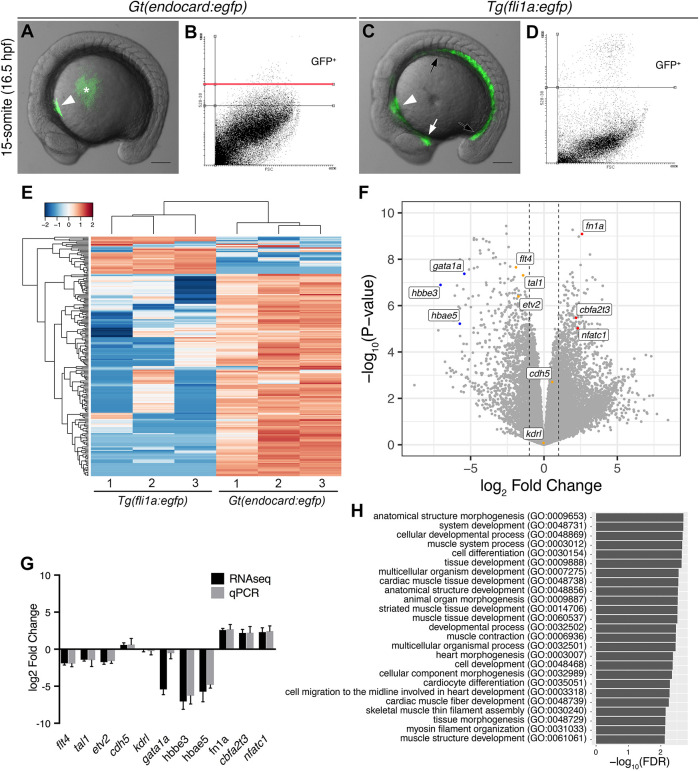
**The endocardium is molecularly distinct from the vascular endothelium.** (A,C) Live images of *Gt(endocard:egfp)* and *Tg(fli1a:egfp)* embryos at the 15 somite-stage (16.5 hpf) showing GFP fluorescence. GFP fluorescence can be seen in the endocardial progenitors (white arrowhead) of *Gt(endocard:egfp)* embryos (A), some auto-fluorescence is seen in the yolk (asterisk). In *Tg(fli1a:egfp)* embryos (C), GFP fluorescence can be seen in the entire vascular endothelium of the trunk (black arrows) and head (white arrow) as well as the endocardium (white arrowhead). Scale bars: 50 μm. (B,D) FACS plots from dissociated *Gt(endocard:egfp)* (B) and *Tg(fli1a:egfp)* (D) embryos show the gating strategy to collect GFP^+^ cells: two gates were used to collect high and low GFP^+^ populations for *Gt(endocard:egfp)* embryos, and all analysis was performed on cells collected above the red line (B), whereas a single population was collected for *Tg(fli1a:egfp)* embryos (D). (E) Hierarchical clustering of the top 200 most variable genes across all samples shows that samples segregate according to transgene. (F) Volcano plot showing the spread of differentially expressed genes with a subset of genes labelled, the dotted lines show a log_2_ fold change of 1 or −1. (G) Validation of the subset of genes highlighted in F by qPCR (G). Log_2_ fold change is represented, error bars indicate 95% confidence intervals. (H) GO term analysis using a PANTHER overrepresentation test was performed on TREAT lists of genes upregulated in the *Gt(endocard:egfp)^High^* samples relative to *Tg(fli1a:egfp)*. The 25 most significant GO terms for genes enriched in the endocardium are shown.

Given that at early stages *Gt(endocard:egfp)* expression is enriched in the endocardium, we used this line to compare the transcriptome of the endocardium with *fli1a*:eGFP-positive cells (primarily endothelium and blood cells). At 15 s, GFP cells were isolated from *Gt(endocard:egfp)* and *Tg(fli1a:egfp)* embryos using fluorescence-activated cell sorting (FACS) (Fig. 2A-D) and mRNA-sequencing performed. Principal component analysis showed that endocardial samples clustered separately from endothelial samples (Fig. S3A). To confirm the integration site of the *Gt(endocard:egfp)* line, RNA-sequencing reads were mapped to the gene-trap cassette. These reads extended into exon 1 of the *map3k22* gene, confirming the reported integration site (Fig. S3B) (Bussmann et al., 2010). Despite mapping to this locus, *in situ* hybridisation (ISH) analysis of *map3k22* showed no detectable expression in the endocardial region (S.J.C., unpublished). Furthermore, no *map3k22* transcripts were detected in the *Tg(fli1a:egfp)* transcriptome. This suggests that rather than the *Gt(SAGFF27C)* gene-trap line reporting bona fide *map3k22* expression, the endocardial expression observed is an artefact either of chromosomal rearrangement from the particular gene-trap insertion event and/or is reporting expression from a nearby locus. Whichever is the case, we have not determined it here.

Hierarchical clustering of the top 200 most variable genes showed that endocardial and endothelial samples separate according to cell type (Fig. 2E). Differential expression analysis found a total of 122 statistically significant genes enriched in the endocardium and 165 enriched in the endothelium (Fig. 2F). To validate these differences, a subset of genes representing blood, endothelium and endocardium were validated by qPCR (Fig. 2F,G). Genes related to red blood cell development, *gata1a*, *hbbe3* and *hbae5*, were enriched in the *fli1a:egfp^+^* population as expected due to these cells arising from the intermediate cell mass in the trunk of the embryo (Davidson and Zon, 2004) (Fig. 2F), although we were unable to validate the *gata1a* expression by qPCR (Fig. 2G). Surprisingly, of the expected pan-endothelial markers analysed, only *kdrl* and *cdh5* were found to show no statistically significant fold change, whereas *flt4*, *scl* and *etv2* were all significantly enriched in the endothelium (Fig. 2F). Reassuringly, *fn1a* and *nfatc1*, markers previously reported to be expressed in the endocardium (Palencia-Desai et al., 2011; Trinh and Stainier, 2004; Wong et al., 2012), were enriched in the *Gt(endocard:egfp)* population (Fig. 2F,G), and GO term analysis on the endocardially enriched gene list retrieved terms relating to heart development and morphogenesis (Fig. 2H; Table S1). To identify genes with previously unreported expression in the endocardium, several were selected for ISH-based screening to validate endocardial expression (Fig. S4). Of these, a subset was found to have restricted expression in regions consistent with the endocardium, including *cbfa2t3*, *gfi1ab*, *hapln1b* and *nrp1a* (Fig. 2; Fig. S4).

**Fig. 3.**
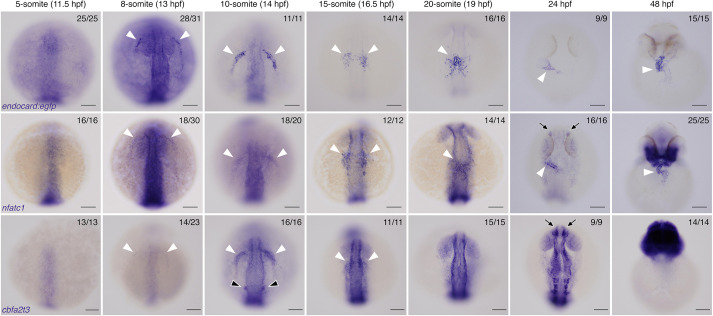
**Endocardial markers are first expressed at 8 s in zebrafish embryos.** *In situ* hybridisation for *endocard:egfp*, *nfatc1* and *cbfa2t3* expression in embryos from 5 s (11.5 hpf) through to 48 hpf, showing expression emerging in the endocardium from 8 s onwards. White arrowheads show the expression domains corresponding to endocardial cells and their progenitors. *nfatc1* expression is also observed in the olfactory placode (black arrows) as previously described. *cbfa2t3* expression is also seen in otic vesicle progenitors (black arrowheads) and throughout the neural system (black arrows). Anterior to the top in all images. Anterior views at 48 hpf, all other images show dorsal views. Scale bars: 100 μm. The number of embryos matching the image shown is indicated in the top right of each image.

To investigate the earliest time point at which endocardial progenitors begin to express these markers, expression of the *Gt(endocard:egfp)* line was examined by ISH over a developmental time course from 5 s (11.5 hpf) through to 48 hpf (Fig. 3). *endocard:egfp* expression was first detected as early as 8 s (13 hpf). This expression was observed in faint semilunar domains in the lateral regions at the anterior of the embryo (Fig. 3) and were lost in an *npas4l* mutant (Fig. S5). These bilateral expression domains were observed more caudal at 10 s (Fig. 3). By 15 s (16.5 hpf), expression was found at the midline in the region of the endocardial core of the cardiac disc (Fig. 3), consistent with the observations made by examining fluorescence (Fig. 1). As *nfatc1* expression was also found to be enriched in the endocardial population at 15 s (Fig. 2F,G), its expression was also analysed over the same time course. *nfatc1* expression was similarly found to initiate as early as 8 s and was observed in domains comparable with that of *endocard:egfp* expression, although much weaker (Fig. 3). Finally, *cbfa2t3* (identified from transcriptome analysis; Fig. 2; Fig. S4) was analysed over the same time course. *cbfa2t3* expression was also found to initiate at 8 s, although at very weak levels, and was observed in similar domains up until at least 15 s (Fig. 3). By 20 s, *cbfa2t3* expression was no longer apparent in the endocardium, although expression continued in the otic placode, olfactory placode, nervous system and head (Fig. 3). Together, these results show that the endocardium expresses multiple unique markers as early as 8 s, much earlier than previously appreciated in zebrafish development.

### *npas4l* and *etv2*, but not *scl*, are required for endocardial progenitor commitment

Previous studies have elegantly demonstrated that perturbation of fate-determining transcription factors can impact the expression domains of myocardial, endothelial and myeloid populations across the ALPM (Keegan et al., 2005; Lieschke et al., 2002; Palencia-Desai et al., 2011; Schoenebeck et al., 2007; Simões et al., 2011; Sumanas et al., 2008). However, without a unique marker of endocardial identity, this population is yet to be studied. Having established the *Gt(endocard:egfp)* line as a marker of the endocardium, we first performed expression analysis to confirm its location in relation to these other well-studied cell populations (Fig. S6). Double fluorescent ISH analysis of the myocardial marker *myl7*, combined with *egfp* expression, at 15 s showed endocardial progenitors located rostral and medial to myocardial progenitors (Fig. S6). Double fluorescent ISH analysis of the myeloid marker *spi1b* and *egfp* showed endocardium located caudal and medial to myeloid cells (Fig. S6). Combining this location with expression of *Gt(endocard:rfp)* on the *Tg(kdrl:egfp)* background at 20 s allowed us to make a map of the endocardial expression domain in relation to these other developing primordia (Fig. S6). Importantly, expression of each of these markers [with the exception of *Gt(endocard:rfp)* and *Tg(kdrl:egfp)*] showed mutually exclusive expression at 15 s.

**Fig. 4.**
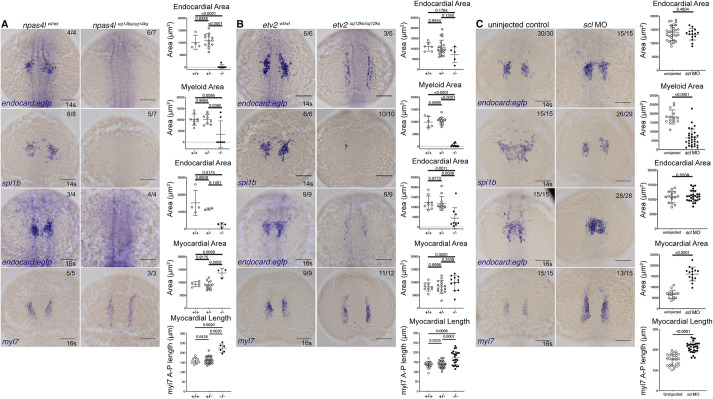
***Gt(endocard:egfp)* expression is reduced in *npas4l* and *etv2* mutants.** (A-C) *In situ* hybridisation for *endocard:egfp* and *spi1b* expression at 14 s and *endocard:egfp* and *myl7* expression at the 16 s in *npas4l^uq14ks^* mutants (A), *etv2^uq13ks^* mutants (B) and *scl* morphants (C). Quantification of the expression domain was measured by calculating the surface area of the stain or the length of the staining domain. These measurements are shown in adjacent graphs, where wild-type (+/+), heterozygous (+/−) and mutant (−/−) quantification is depicted for *npas4l^uq14ks^* and *etv2^uq13ks^* mutants and siblings, and uninjected or morphant treatment for *scl*. Dorsal views, with anterior to the top. Scale bars: 100 μm. Data are mean±s.d. *P*-values are present in graphs were calculated using a one-way ANOVA (for *npas4l* and *etv2* mutant analysis) or an unpaired two-tailed *t*-test (for *scl* MO analysis).

Next, we generated loss-of-function models for key regulators of endothelial identity and investigated their effect on endocardial development. CRISPR/Cas9 genome-editing was used to create loss-of-function mutants for *npas4l* and *etv2* genes (Fig. S7) and a previously validated morpholino against *scl* was used to deplete *scl*. Each of these models was phenotypically characterised and found to phenocopy published loss-of-function models (Fig. 4; Fig. S7). These models were examined for endocardial (*endocard:egfp)*, myeloid (*spi1b*) and myocardial (*myl7*) expression.

As expected, endocardial expression was completely absent in *npas4l* mutants at both 14 s and 16 s (Fig. 4A). The myeloid expression domain was also completely absent in mutants, consistent with previous reports (Lieschke et al., 2002). The myocardial expression domains were expanded (Fig. 4A), as described previously (Schoenebeck et al., 2007).

We next examined *etv2* mutants and found a trend toward decreased staining area for endocardial expression at 14 s (Fig. 4B). Most notably, the bilateral *endocard:egfp* expression in these mutants was extended along the anterior-posterior axis and localised to the lateral margins of the embryo (consistent with vascular endothelial localisation), suggesting presumptive endocardium is failing to migrate to the midline as in wild-type siblings (Fig. 4B). By 16 s, *endocard:egfp* expression is significantly reduced and cell counts show a dramatic 98% reduction in cell numbers, compared with sibling controls (Fig. S7). In addition, *spi1b* expression is lost at 14 s and *myl7* expression expanded in length at 16 s (Fig. 4B).

Surprisingly, *scl* knockdown had little effect on the expression domain of *endocard:egfp* (Fig. 4C). This contrasts with cell counts for *scl* morphants, which showed a small (25%) reduction in cell number (Fig. S7). Interestingly, these data signify a clear distinction between *etv2* and *scl* function in this endothelial population. At 16 s, the spatial organisation of endocardial expression was altered in *scl* morphants, in accordance with a previously described role for *scl* in endocardial cell migration and morphology (Bussmann et al., 2007; Schumacher et al., 2013). Also consistent with previous reports (Gering et al., 1998; Patterson et al., 2005; Shivdasani et al., 1995), a significant reduction of the myeloid domain was observed (Fig. 4C) as well as an increased myocardial expression domain.

Together, these results show that transcription factors *npas4l* and *etv2* are required for endocardial development. Furthermore, *scl* is dispensable for early endocardial differentiation, despite its previously described role in endocardial morphogenesis (Bussmann et al., 2007; Schumacher et al., 2013) and maintenance of endocardial identity during later development (Clay and Ramakrishnan, 2005; Schumacher et al., 2013; Van Handel et al., 2012).

### Bmp signalling is required for endocardial differentiation

**Fig. 5.**
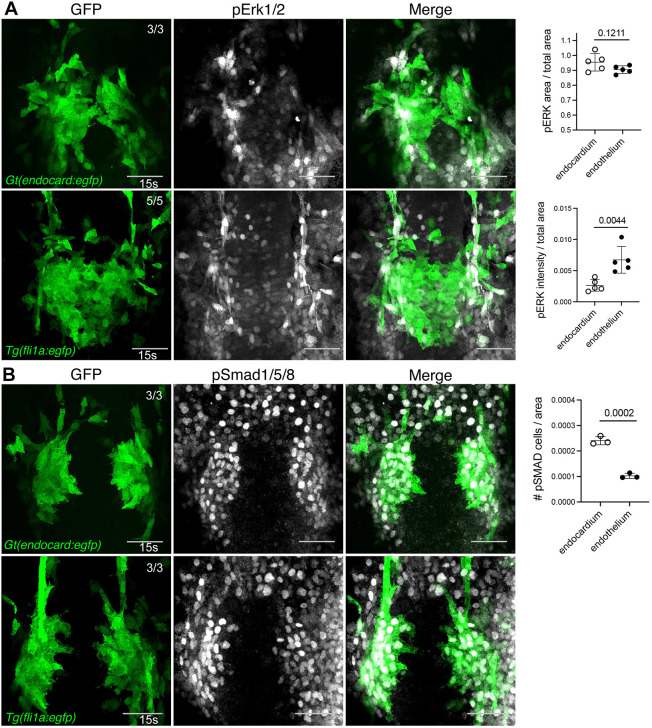
**Bmp signalling is active in developing endocardial cells.** (A,B) Immunofluorescence staining for pErk1/2 (A) in *Gt(endocard:egfp)* and *Tg(fli1a:egfp)* embryos at 15 s shows minimal pErk1/2 signal in endocardial cells and high activity in the adjacent vascular endothelium. By contrast, pSmad1/5/8 (B) in *Gt(endocard:egfp)* and *Tg(fli1a:egfp)* embryos at 15 s shows high pSmad1/5/8 activity in developing endocardial cells but minimal activity in adjacent vasculature. Quantification of pErk1/2 area and intensity or pSMAD cell number in endocardium or adjacent vasculature is depicted in neighbouring graphs. Dorsal views are shown with anterior to the top in all images. Scale bars: 50 μm. Data are mean±s.d. *P*-values are present in graphs (determined by an unpaired two-tailed *t*-test).

Next, we examined downstream effectors of two signalling pathways known to be important for cardiovascular development. In zebrafish, VEGF signalling has previously been shown to act through Erk during early stages of angiogenesis (Shin et al., 2016a,b) and Bmp signalling through Smad1/5/8 (Derynck and Zhang, 2003). Analysing phosphorylated Erk 1/2 (pErk) and phosphorylated Smad1/5/8 (pSmad) in *Gt(endocard:egfp)* or *Tg(fli1a:egfp)* embryos at 15 s showed considerable co-labelling of pSmad with endocardial progenitors, whereas pErk labelling was observed predominantly in adjacent vascular endothelium (Fig. 5A). Quantification confirmed this, showing increased pErk intensity in endothelium compared with endocardial progenitors (Fig. 5; Fig. S8). Conversely, an increase in the number of pSmad-positive cells was observed in endocardial progenitors compared with adjacent vascular endothelium. This suggests that Bmp signalling is more active in endocardium than developing vasculature, whereas Erk is more active in vasculature than endocardium.

**Fig. 6.**
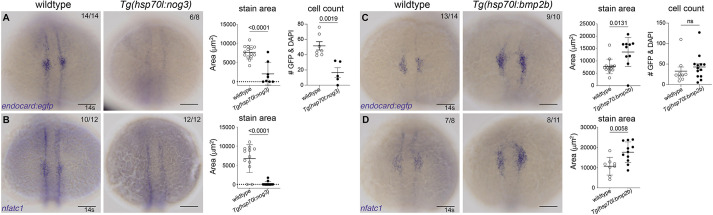
**Bmp signalling is required for endocardial development.** (A,B) *In situ* hybridisation for endocardial markers *endocard:egfp* (A) and *nfatc1* (B) in wild-type (transgenic negative siblings) or *Tg(hsp70l:nog3)* embryos at the 14 somite (s) stage [heat-shock performed at tailbud stage (10 hpf)] shows significantly reduced staining of the endocardial domain upon inhibition of Bmp signalling. Quantification of the staining area of expression is shown in adjacent graphs. Quantification of GFP-positive and DAPI-positive cell number is also shown, as determined by confocal imaging of *Gt(endocard:egfp)*, DAPI-stained embryos at 14-15 s. (C,D) Wild-type sibling controls or *Tg(hsp70l:bmp2b)* embryos at 14 s (heat-shocked at tailbud) show increased staining area in embryos stained for *endocard:egfp* (C) or *nfatc1* (D). Quantification of the area of expression shown in adjacent graphs. Dorsal views are shown with anterior to the top in all images. Scale bars: 100 μm. Data are mean±s.e.m. *P*-values are present in graphs (unpaired two-tailed *t*-test). ns, not significant.

To investigate whether Bmp signalling plays a functional role in endocardial development, the *Gt(endocard:egfp)* line was crossed with the heat-shock-inducible transgenic lines, *Tg(hsp70l:bmp2b)* and *Tg(hsp70l:nog3)* (Chocron et al., 2007), either activating or inhibiting the Bmp pathway, respectively. Embryos were heat-shocked at tailbud stage (10 hpf) and fixed at 14 s or 16 s for analysis of the myeloid, endocardial and myocardial domains. Inhibition of the Bmp signalling pathway by global induction of *nog3* expression resulted in a reduction of endocardial (Fig. 6), myeloid and myocardial expression domains at 14 s and 16 s (Fig. S9). Reciprocally, activation of the Bmp signalling pathway by global induction of *bmp2b* expression caused a significant expansion of the endocardial (Fig. 6) and myeloid (Fig. S9) expression domains but did not increase in the myocardial domain area (Fig. S9). Interestingly, activating Bmp signalling not only increased the endocardial expression domain but also resulted in stronger staining compared with siblings lacking the heat-shock transgene (Fig. 6). Quantification of cell number showed a trend but no significant increase in cell number following increased Bmp signalling, implying the expansion of the endocardial domain is through increased transcription of *egfp* within endocardial cells. These results show that Bmp signalling is necessary for early endocardial development.

### Bmp signals through *npas4l* and *etv2* to instruct endocardial differentiation

Given the dependency of the endocardium on *npas4l* and *etv2* as well as Bmp signalling (Figs 4, 6; Fig. S9), we examined whether *npas4l* and *etv2* expression were perturbed by altered Bmp signalling. Inhibition of Bmp signalling by heat-shock at the tailbud stage did not abolish either *npas4l* or *etv2* expression but significantly reduced the expression of *npas4l* and altered patterning of *etv2* (Fig. S10). In addition, weak *etv2* expression could be detected by ISH in the endocardium at 20 s. This expression was reduced or lost upon inhibition of Bmp signalling (Fig. S10), suggesting that Bmp signalling is required for *etv2* expression.

**Fig. 7.**
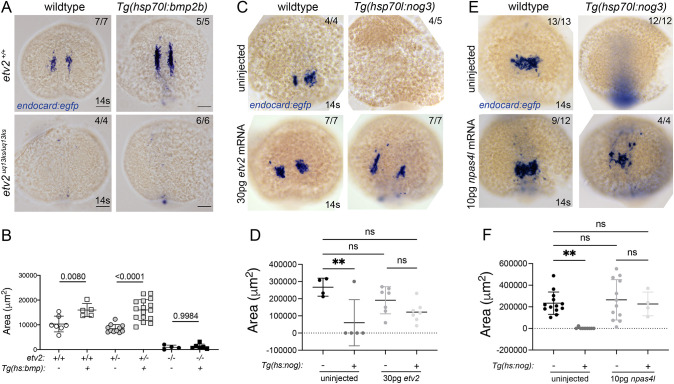
**Epistasis experiments show that *npas4l* and *etv2* are downstream of Bmp signalling.** (A) *In situ* hybridisation for *endocard:egfp* expression in wild type and *etv2* mutant with and without the *hsp70l:bmp2b* transgene. *etv2* mutants have reduced *endocard:egfp* expression, irrespective of Bmp overexpression. (B) Quantification of the staining area shows Bmp overexpression increased the *endocard:egfp* expression domain whereas *endocard:egfp* expression is lost in *etv2* mutants, regardless of Bmp overexpression. (C-F) Reciprocal experiments involving *in situ* hybridisation for *endocard:egfp* expression in wild-type (transgenic negative siblings) or *Tg(hsp70l:nog3)* embryos injected with 30 pg of *etv2* (C) or 10 pg of *npas4l* (E) mRNA. Quantification of staining area shows that both *etv2* (D) and *npas4l* (F) mRNA overexpression can restore *endocard:egfp* expression upon Bmp inhibition. Scale bars: 100 μm. Data are mean±s.d. *P*-values are indicated in graphs. **P<0.01 (one-way ANOVA). ns, not significant.

We next examined the epistatic relationship between Bmp signalling and *etv2* in endocardial development. Heat-shock of *Tg(hsp70l:bmp2b)* at tailbud stage had no effect on endocardial expression in the absence of *etv2*, in that overactivation of Bmp signalling could not rescue the lost endocardial expression observed in *etv2* mutants (Fig. 7). This suggests that *etv2* functions downstream of Bmp signalling to induce endocardial differentiation. To prove this, the reciprocal experiment was performed, overexpressing *etv2* in *Tg(hsp70l:nog3)* embryos (Fig. S11). Although endocardial expression was reduced upon inhibition of Bmp signalling compared with the wild type (Fig. 6), overexpression of *etv2* upon inhibition of Bmp signalling was capable of restoring *endocard:egfp* expression (Fig. 7). A similar phenomenon was observed for *npas4l* overexpression in Bmp-inhibited embryos, suggesting that *etv2* and *npas4l* are sufficient for endocardial differentiation in the absence of Bmp signalling. Together, these results demonstrate that *npas4l* and *etv2* function downstream of Bmp signalling to induce endocardial differentiation.

## DISCUSSION

The endocardium is a unique invention of the vertebrate taxa (Perez-Pomares et al., 2009). Although the endocardium has many molecular similarities to the adjacent vascular endothelium, there are distinct physiological functions of these tissues (Harris and Black, 2010). What distinguishes these lineages on a molecular level remains an important and underexplored question in cardiovascular biology.

Characterisation of the *Gt(endocard:egfp)* line during somitogenesis stages shows expression of this marker enriched in endocardial progenitors as early as 8 s. Importantly, we found that *nfatc1* is expressed in similar spatiotemporal domains and a newly identified marker, *cbfa2t3*, also has similar expression. These results show that the endocardium is specified earlier than previously appreciated in zebrafish development and that a unique transcriptional profile is established before the formation of the linear heart tube, bringing it in line with what has been reported in mice (de la Pompa et al., 1998). The observed onset of endocardial expression at 8 s is suggestive that endocardial identity is assumed close to this time in development. This marries well with observations of *npas4l* and *etv2* (*etv2* is a direct target of *npas4l*) expression – upstream regulators of endothelial identity. *npas4l* expression is reported to peak at tailbud stages and remain high until 15 s, whereas *etv2* initiates weakly at tailbud and is highly expressed by 15 s (Reischauer et al., 2016). The expression of these two transcription factors that are required for endocardial development is consistent with the onset of endocardial identity from 8 s.

The 8 s stage represents an intriguing time point for endothelial fate decisions. In *scl* morphants, the mesodermal markers *hhex* and *draculin* are substantially reduced from 7 s, despite initiating expression normally at earlier stages (Patterson et al., 2005). These results suggest that this stage marks a crucial time point in development at which endothelial progenitors undergo significant changes, perhaps committing to specialised lineages, such as the endocardium. Adding to this intrigue, both *snai1b* and *myod1* expression in the forming somites undergo unique changes at 7 s (Hammerschmidt and Nusslein-Volhard, 1993; Weinberg et al., 1996), suggesting that such fate decisions may occur more broadly across the mesoderm around this stage.

Surprisingly, we found that *scl* morphants had only minor defects in early endocardial development despite its important role in blood development, a role in endocardial morphogenesis (Bussmann et al., 2007; Schumacher et al., 2013) and a later maintenance role in endocardial identity (Schumacher et al., 2013; Van Handel et al., 2012). We therefore propose that, although *scl* is an important transcription factor in endocardial development, it is in fact dispensable for the early differentiation of the endocardial lineage. Unlike *scl*, we found that *etv2* is required for endocardial differentiation. Similar observations have been made in mouse, with *Etv2* knockout resulting in an absence of endocardium (Ferdous et al., 2009). This suggests a conserved role for *Etv2* in endocardial development.

In addition to our analysis of transcription factors, we also report the requirement for Bmp signalling in endocardial differentiation. Bmp signalling has previously been implicated in endocardial development, particularly in the formation of the cardiac valves (Dietrich et al., 2014; Misfeldt et al., 2009; Saxon et al., 2017; Snider et al., 2014; Wang et al., 2005) and in paracrine signalling between the endocardium and myocardium (Palencia-Desai et al., 2015; Saint-Jean et al., 2019). More broadly in cardiac development, it has also been shown to regulate cardiomyocyte differentiation in the cardiac disc (de Pater et al., 2012) and to instruct cardiac progenitor cell migration during linear heart tube formation (Smith et al., 2008). The successive and yet diverse roles for the Bmp pathway in cardiac development make it challenging to investigate. For example, somewhat counterintuitive in this study, we observe enriched pSmad in the endocardium after it has differentiated (Fig. 5B), consistent with previous studies indicating an ongoing requirement for Bmp signalling after differentiation has occurred (Palencia-Desai et al., 2015; Snider et al., 2014). It is feasible and perhaps likely that Bmp signalling plays additional roles in endocardial development, and possibly also endothelial development, that have yet to be described. Whether this is the case or not, we provide strong evidence here, through expression studies and epistasis experimentation, that *npas4l* and *etv2* function downstream of Bmp signalling in endocardial differentiation. Consistent with this, *Etv2* has been reported to function downstream of Bmp signalling in the generation of *kdrl*^+^ mesoderm *in vitro* (Lee et al., 2008) and in the posterior lateral plate mesoderm in the zebrafish (Row et al., 2018). Furthermore, characterisation of the murine *Etv2* promoter has identified multiple SMAD binding sites (Shi et al., 2015) suggesting direct regulation of *Etv2* by Bmp may occur. Intriguingly, *npas4l* was recently shown to bind to the *etv2* promoter and regulate its expression (Marass et al., 2019) and we find here that *npas4l* overexpression is capable of inducing endocardial differentiation despite Bmp inhibition. This suggests that SMAD cooperativity is not required for *npas4l* activity in endocardial development. Together, these findings suggest that Bmp signalling, upstream of *npas4l* and *etv2*, plays a crucial role in regulating endocardial development.

Finally, further characterisation of the endocardium by RNA-sequencing demonstrates a unique molecular signature distinguishing the endocardium from vascular endothelium. These results are in agreement with an increasing number of single cell RNA-sequencing studies from human, mouse, chick and zebrafish, showing the separation of endocardial cells from other endothelial populations. Importantly, many of these studies include genes such as *nfatc1*, *cbfat2t3* and *itga9* (and others identified and confirmed via ISH; Fig. S4) as expressed in the endocardial cell population, supporting the notion of a conserved regulatory programme for endocardial development (Chestnut et al., 2020; Hu et al., 2018; Ma et al., 2021; Mantri et al., 2021; Spanjaard et al., 2018; Tucker et al., 2020). This suggests that further analysis of the endocardial transcriptome in model organisms from equivalent stages may identify novel endocardial regulators. Given that the genetic aetiology of many congenital heart disease patients remains unclear (Morton et al., 2022), functional analysis of such regulators is required to improve clinical outcomes. Further, a number of studies have reported the differentiation of endocardial-like cells (Hofbauer et al., 2021; Mikryukov et al., 2021; Neri et al., 2019; Palpant et al., 2015), yet how closely these mimic *in vivo*-derived endocardium remains unclear, in part because of a lack of molecular markers for such a comparison. We believe that the identification of a core subset of conserved endocardial regulators will further aid attempts to generate endocardial tissue and model cardiac development *in vitro*.

## MATERIALS AND METHODS

### Zebrafish lines

All zebrafish strains were maintained and animal work performed in accordance with the guidelines of the animal ethics committee at The University of Queensland and The University of Melbourne, Australia. The previously published transgenic lines used in this study are *Gt(SAGFF27C); Tg(4xuas:gfp)*, referred to as *Gt(endocard:egfp)* in this text (Bussmann et al., 2010), *Tg(kdrl:Hsa.HRAS-mCherry)^s916^* (Hogan et al., 2009), *Tg(fli1a:egfp)^y1^* (Lawson and Weinstein, 2002), *Tg(hsp70l:nog3)^fr14^* and *Tg(hsp70l:Bmp2b)^fr13^* (Chocron et al., 2007), *Tg(kdrl:egfp)^s843^* (Jin et al., 2005). The construct for the previously published *Tg(myl7:mCherry-CaaX)^bns7^* line (Uribe et al., 2018) was injected to establish the *uqks35* allele (Koopman et al., 2021).

### Antibody staining

Phospho-protein staining was performed as previously described (De Angelis et al., 2017; Okuda et al., 2018) using the following antibodies: GFP (Abcam, ab13970, 1:500), pErk1/2 (Cell Signaling Technology, 4370, 1:250), pSmad1/5/8 (Cell Signaling Technology, 13820, 1:250), DsRed (Living Colors DsRed Polyclonal Antibody, Takara, 623496, 1:500) as previously described (Grassini et al., 2019). Two rounds of independent staining were performed for each pErk1/2 and pSmad1/5/8 stain and *n* is depicted in the images and accompanying dot-plots.

### Fluorescent ISH

Fluorescent ISH was performed as previously described (Baek et al., 2019; Brend and Holley, 2009; Clay and Ramakrishnan, 2005) with minor alterations. In brief, embryos were dechorionated and fixed at the desired stage. RNA probes were synthesised with DIG or FLU RNA labelling mix (Roche). Fixed embryos were permeabilised with proteinase K (Invitrogen) and hybridised with 1 ng/μl RNA probe in hybridisation buffer overnight at 70°C. After hybridisation, embryos were washed, blocked with western blocking reagent (Roche) and incubated with anti-dig or anti-flu, POD antibodies (Roche) in western blocking reagent overnight at 4°C. After further washes, staining was performed using the tyramide signalling amplification kit (Perkin Elmer) for 2 h at 37°C. Following staining, embryos were fixed at 4°C overnight and mounted for imaging.

### FACS and RNA-sequencing

To prepare for FACS, an outcross of either homozygous *Gt(endocard:egfp)* or heterozygous *Tg(fli1a:egfp)* fish to wild-type fish was set up and embryos collected 30 min after dividers were removed. Fertilised embryos were kept at 28.5°C until they reached gastrulation stages, at which point they were placed at 23°C to slow down development. The following morning, embryos were examined to determine their developmental stage according to the number of somites present before being returned to 28.5°C. Immediately before the 15 s stage, embryos were dechorionated by adding pronase. At the 15 s stage, embryos were collected and dissociated for FACS. Briefly, embryos were de-yolked by pipetting embryos in calcium-free Ringer's solution. Embryos were then centrifuged at 2000 rpm (∼400 ***g***) for 5 min at 4°C, the supernatant removed and replaced with protease solution [a 1:35 dilution of 2.5 mg/ml Liberase TM (Roche) in PBS]. Embryos were then dissociated by incubating at 28.5°C for 10 min with intermittent homogenisation by pipette. Dissociation was stopped by placing samples on ice and adding CaCl_2_ and foetal calf serum to final concentrations of 2 mM and 10%, respectively. Samples were then centrifuged at 2000 rpm for 5 min at 4°C, the supernatant removed and the resulting cell pellet resuspended in PBS with 2 mM EDTA and sorted at Queensland Brain Institute's flow cytometry facility. Only high-expressing cells were collected and used for further analysis, to limit the contribution of cardiomyocytes that express eGFP weakly. GFP+ cells were collected in TRIzol LS reagent (Thermo Fisher Scientific) and RNA extracted using Direct-zol RNA MiniPrep columns (Zymo Research). As the total amount of RNA recovered from these samples was low, RNA was amplified using the Smart-seq2 protocol (Picelli et al., 2014). Briefly, 2 ng of RNA was reverse transcribed using SuperScript II reverse transcriptase (Thermo Fisher Scientific) with oligo-dT and template switching oligo primers. The resulting cDNA was then amplified using KAPA HiFi HotStart ReadyMix (Roche) and the ISPCR primer. The resulting amplified cDNA was then purified using the Axygen AxyPrep Mag Fragment Select Kit (Thermo Fisher Scientific). Illumina library preparation, sequencing and analysis was performed by the University of Queensland Institute for Molecular Bioscience (IMB) Sequencing Facility.

### Read mapping, counting and differential expression analysis

All analysis was performed using RStudio (http://www.rstudio.com/). Reads were mapped to the GRCz11 version of the zebrafish genome downloaded from Ensembl using the Rsubread package (Liao et al., 2019). Counting of reads was also performed using Rsubread. Differential expression analysis was performed using the limma package (Law et al., 2014; Ritchie et al., 2015). Figures were produced with the ggplot2 package (Wickham, 2009).

### qPCR

qPCR was performed on amplified RNA samples prepared as described above using the Smart-seq2 protocol (Picelli et al., 2014). Amplified cDNA was diluted 1:80 and used in a reaction with SYBR Green (Thermo Fisher Scientific) and primers specific to the gene of interest. Each gene was analysed in triplicate for each sample. Amplification efficiencies for each primer pair were calculated using LineRegPCR (Ramakers et al., 2003). Four housekeeping genes were examined to identify the most stable gene using RefFinder (Xie et al., 2012) and the most stable gene used to normalise samples with Q-Gene (Muller et al., 2002; Simon, 2003).

### GO term analysis

To examine GO terms exclusively associated with the endocardium and vascular endothelium, an overrepresentation test was performed using the PANTHER database available at http://pantherdb.org/ (Mi et al., 2019). Upregulated and downregulated gene lists were filtered from differentially expressed genes with a log_2_ fold change cut-off of ≥1 and ≤−1 and an adjusted *P*-value of ≤0.05, applied using TREAT (McCarthy and Smyth, 2009) in RStudio (http://www.rstudio.com/).

### ISH

ISH was performed as previously described (Grassini et al., 2018). All RNA probes but one were transcribed from plasmids. Plasmids containing the *spi1b* and *gfp* coding sequences were a kind gift from the laboratory of Ben Hogan (Peter MacCallum Cancer Research Institute, Melbourne, Australia). To generate the *etv2* plasmid used in this study, pCS2+ vector was prepared by digesting empty vector with BamHI and XbaI. The *etv2* cDNA sequence was amplified by PCR using the primer sequences: *etv2*_cDNA_F, AAGCTACTTGTTCTTTTTGCAGGATCTGTCAAAACCCCTGATATAGTG; *etv2*_cDNA_R, TGGATCTACGTAATACGACTCACTATAGTTCTAGCAATCTGCTGCAAAGTCC. The PCR product was purified and inserted into the digested pCS2+ vector using circular polymerase extension cloning (Quan and Tian, 2011). For the *npas4l* probe, primers from Reischauer et al., 2016 were used, with the *Sp6* promoter on the R primer to amplify the template from cDNA.

For all *in situ* data, at least two experimental replicates were performed. Biological replicates are indicated in images, depicting the *n* for each condition.

### CRISPR/Cas9 mutagenesis

To generate the mutant lines used in this study CRISPR/Cas9 mutagenesis was performed as previously described (Capon et al., 2017; Gagnon et al., 2014). Embryos and fish were screened for indels by HRMA (Dahlem et al., 2012) and carriers sequenced to identify frame-shift mutations that truncate the protein (Fig. S7). Specific details of each of the mutants generated for this study are below.

The *npas4l* mutant allele carries a 7 bp deletion in the second exon of the *npas4l* gene and is referred to as the *npas4l^uq14ks^* allele. This deletion creates a frame-shift at amino acid 47 (E47D) followed by seven missense amino acids before a premature stop codon. The predicted protein product of this truncated allele partially retains the DNA-binding basic helix-loop-helix domain but lacks the two PAS domains as well as the three transcriptional activation domains (Fig. S7). An incross of fish carrying the *npas4l^uq14ks^* allele produces a mutant phenotype in 25% of the progeny, consisting of a bell-shaped heart and lacking the endocardial layer at 48 hpf, as previously described for the *npas4l* mutant (Stainier et al., 1995).

The *etv2* mutant allele has a 23 bp deletion in exon five of the *etv2* gene and is referred to as the *etv2^uq13ks^* allele. This deletion creates a frame-shift at amino acid 137 (P137R) resulting in the incorporation of 10 missense amino acids before a stop codon. This truncated allele completely lacks the DNA-binding ETS domain (Fig. S7). An incross of fish heterozygous for the *etv2^uq13ks^* allele produces a mutant phenotype in 25% of progeny. This phenotype is characterised by a lack of blood circulation, as previously reported (Pham et al., 2007), collapsed heart at 48 hpf, impaired intersegmental vessel sprouting and extopic expression of vascular markers in skeletal muscle, as previously reported (Chestnut et al., 2020).

### Morpholino oligonucleotide reagents

All morpholino oligonucleotides (MO) were ordered from Genetools. The MO sequences and concentrations used were as follows: 0.8 pmol *scl* – AATGCTCTTACCATCGTTGATTTCA (Dooley et al., 2005); 1 pmol *gata1a* – CTGCAAGTGTAGTATTGAAGATGTC (Galloway et al., 2005); 1.77 pmol *spi1b* – GATATACTGATACTCCATTGGTGGT (Rhodes et al., 2005).

### Heat-shock treatment

All heat-shock experiments were performed by outcrossing heterozygous carriers of either *Tg(hsp70l:nog3)* or *Tg(hsp70l:bmp2b)* to non-heat-shock transgenic lines, generating clutches of embryos with an expected Mendelian ration of 50% transgenic to 50% wild type. Heat-shock was performed at 39°C for 30 min in pre-warmed medium on mixed-genotype clutches of embryos. Following heat-shock, embryos were returned to a 28.5°C incubator until fixation.

### *In vitro* transcription

To produce the *etv2* and *npas4l* RNA for overexpression experiments, plasmids containing the *etv2* or *npas4l* coding sequences were linearised by restriction enzyme digest. Purified linearised plasmid was transcribed with the mMessage mMachine SP6 kit (Thermo Fisher Scientific). RNA was purified with the RNA clean and concentrator kit (Zymo Research). The *npas4l* plasmid was a kind gift from the laboratory of Didier Stainier (Max Planck Institute, Germany).

### Imaging

Embryos from ISH were dehydrated, cleared in Murray's solution (a 2:1 ratio of benzyl benzoate:benzyl alcohol) and imaged using an Olympus BX51 Microscope with Olympus DP70 CCD camera on glass slides with bridged coverslips. For wide-field, bright-field and fluorescence imaging, embryos were mounted in 3% methyl cellulose (Sigma-Aldrich, M0387) and imaged using a Leica M165 FC stereo microscope with a DFC425 C camera.

Confocal imaging was performed on a Zeiss LSM710 FCS, LSM880 with 40× immersive objective with Airyscan detector and Zeiss LSM900 confocal microscope. Live embryos were mounted for confocal imaging using 0.5-1% low-melting agarose (Sigma-Aldrich, A9414) in glass-bottom Petri dishes. Fixed embryos for confocal imaging were de-yolked using lash tools and mounted in vectashield with DAPI (Vector Laboratories, H-1200) on glass-slides with coverslips. Alternatively, the fixed embryos were mounted in 1% agarose and imaged with an immersive 40× objective.

All images were processed using FIJI (Schindelin et al., 2012) and/or Imaris software. For all imaging of embryos involving crosses of *etv2* or *npas4l* mutants, or *Tg(hsp70l:nog3)* or *Tg(hsp70l:bmp2b)* lines, imaging was performed blind and samples genotyped post-imaging to assign genotypes.

Quantification of pErk and pSmad staining (Fig. 5) was generated using a mask in Imaris software. pErk and pSmad signal was quantified within the mask regions. Example masks are represented in Fig. S8.

### Statistical testing

An unpaired two-tailed Student's *t*-test with Welch's correction was performed using Prism software. For datasets containing three or more conditions, one-way ANOVA testing with Tukey's post hoc test for multiple comparisons was performed using Prism software.

## Supplementary Material

Supplementary information

Reviewer comments
