# Supplementary material for: Endocardial identity is established during early somitogenesis by Bmp signalling acting upstream of npas4l and etv2
Source: Development. 2022 May 9;149(9):dev190421. doi: 10.1242/dev.190421 (PMC9148566; doi:10.1242/dev.190421)
Supplement: Supplementary information [file develop-149-190421-s1.pdf]

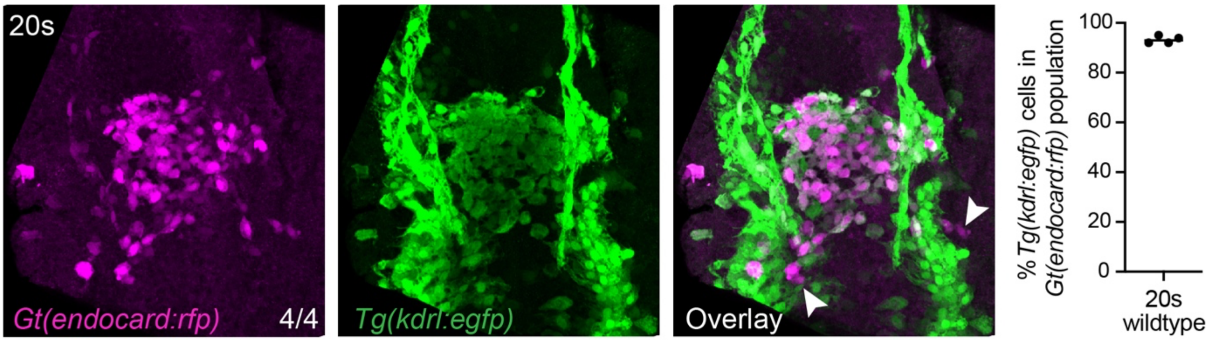

Figure S1

Fig. S1. Quantification of *Tg(kdrl:egfp)* positive cells in the *Gt(endocard:egfp)* population, expressed as a percentage, in 20s stage embryos.

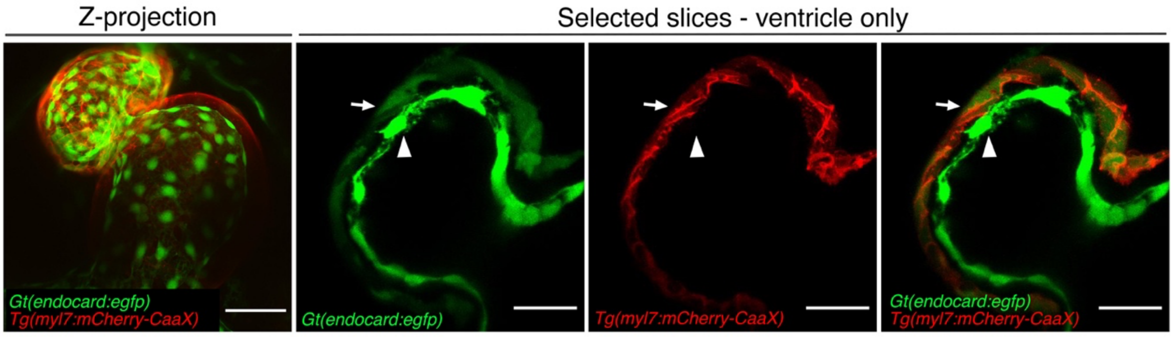

Figure S2

Fig. S2. Live imaging of *Gt(endocard:egfp)*; *Tg(myl7:mCherry-CaaX)* embryos at 48 hpf. A Z-projection of the full heart is shown. Selected slices of the ventricle are also shown demonstrating weak myocardial expression in the *Gt(endocard:egfp)* line. All images are ventral views with anterior to the top. Scale bars represent 50 µm (in Z-projection) and 25 µm (in slices). White arrowheads label endocardium, white arrows label myocardium.

Development • Supplementary information

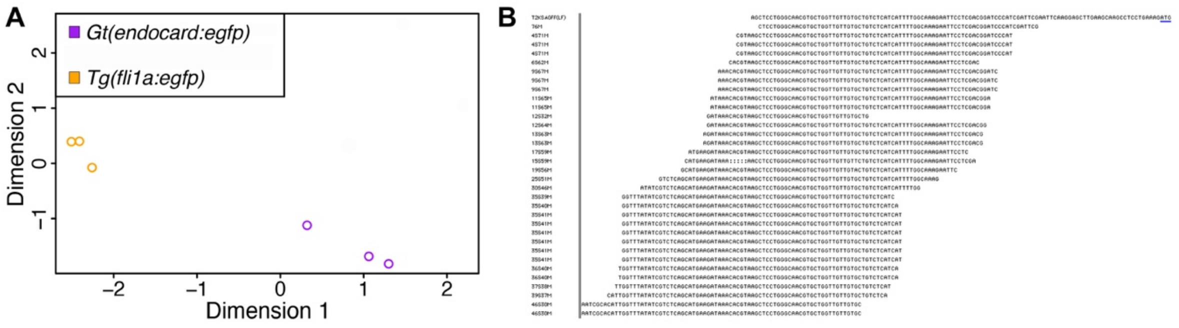

Figure S3

Fig. S3. A. A PCA plot showing the relationship of samples according to dimensions 1 and 2 shows *Gt(endocard:egfp)* and *Tg(fli1a:egfp)* samples form distinct clusters according to both dimensions. **B.** Sequencing reads were mapped to the gene-trap cassette to validate the reported insertion site of the *Gt(endocard:egfp)* line. Soft-clipped reads from *Gt(endocard:egfp)* samples were aligned and the sequence 5’ of the gene-trap cassette blasted against the zebrafish genome. This sequence was found to align perfectly with the 3’ end of the first exon of the *map3k22* gene confirming the insertion of the cassette in the first intron of this gene. The start codon of the GAL4FF coding sequence in the gene-trap cassette is underlined in blue for reference.

Development • Supplementary information

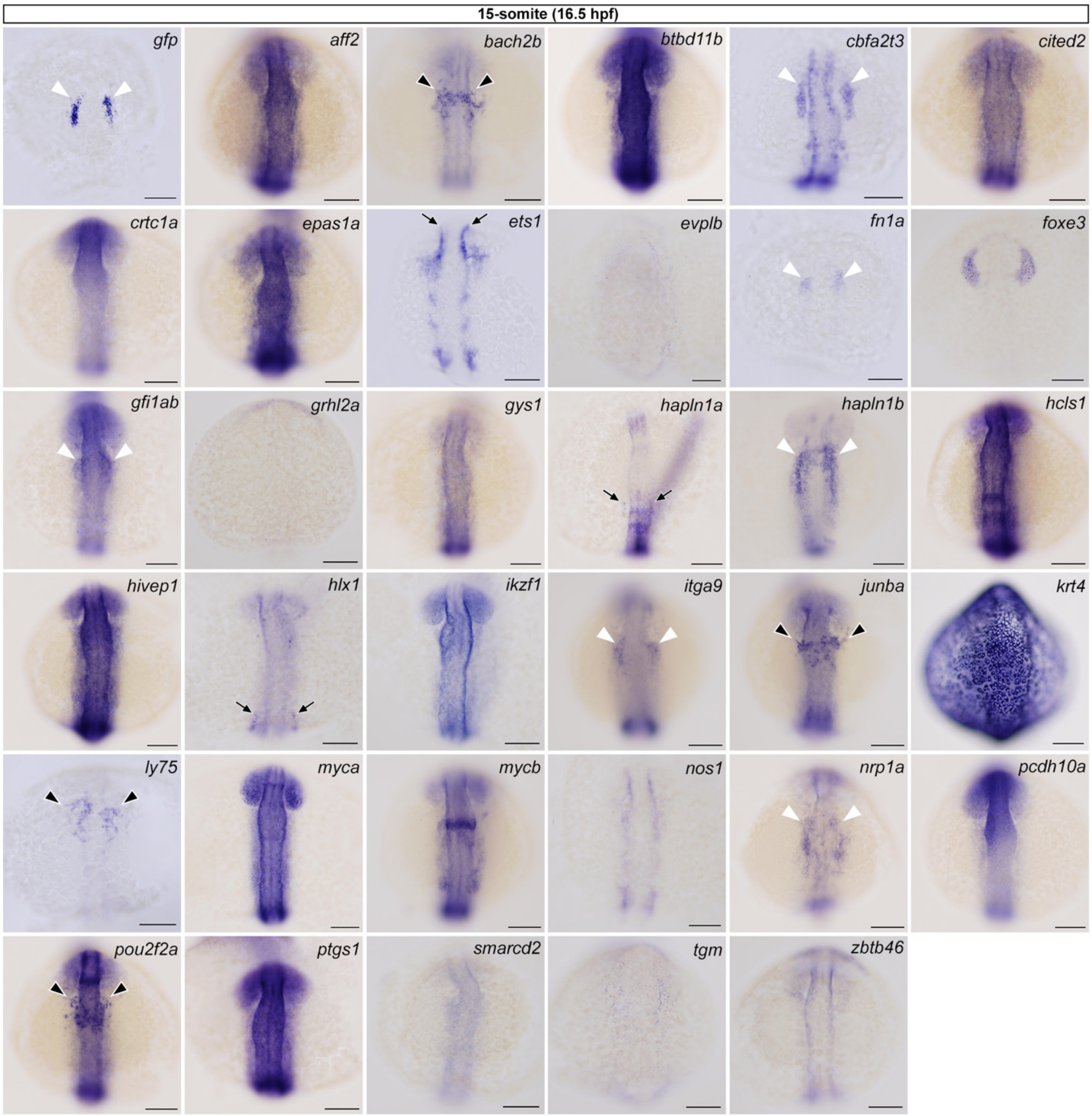

Figure S4

Fig. S4. *In situ* hybridisation for 34 candidate genes showing enrichment in the endocardium in some. All expression patterns were examined at the 15-somite stage (16.5 hpf). As a positive control, a *gfp* probe was examined in *Gt(endocard:egfp)* embryos to label the endocardium. White arrowheads label endocardial domains, black arrowheads label myeloid domains, black arrows label vascular endothelial expression. Ventral views are shown with anterior to the top in all images. Scale bars represent 50µm.

Development • Supplementary information

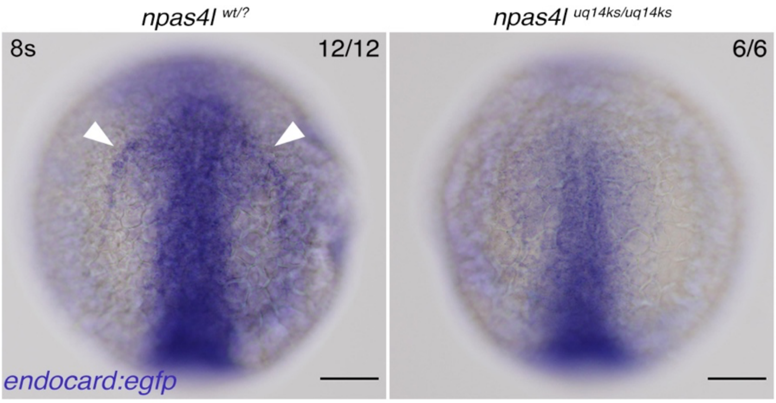

Figure S5

Fig. S5. *In situ* hybridisation for endocardial *gfp* expression in *Gt(endocard:egfp)*; *npas4l* wildtype sibling and homozygous mutant embryos at 8s (13 hpf). Dorsal views with anterior to the top in all images. Scale bars represent 100 µm. The number of embryos matching the image is indicated in the top right of each image.

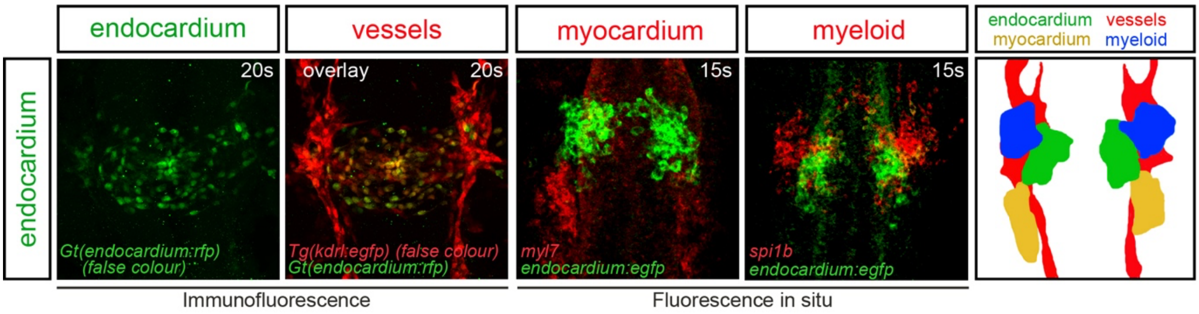

Figure S6

Fig. S6. Expression domains of mesodermal lineages in the mid-somitogenesis stage embryo. Live imaging of *Gt(endocard:rfp)*;*Tg(kdrl:egfp)* embryos at 20s (false coloured). Fluorescent double *in situ* hybridisation *myl7* (red; myocardium) overlaid with *endocardium:egfp* (green; endocardium) and spi1b (red; myeloid) overlaid with *endocardium:egfp* (green; endocardium) at 15s. Schematic representation of spatial arrangement of endocardium, vessels, myocardium and myeloid expression domains in 15-20s.

Development • Supplementary information

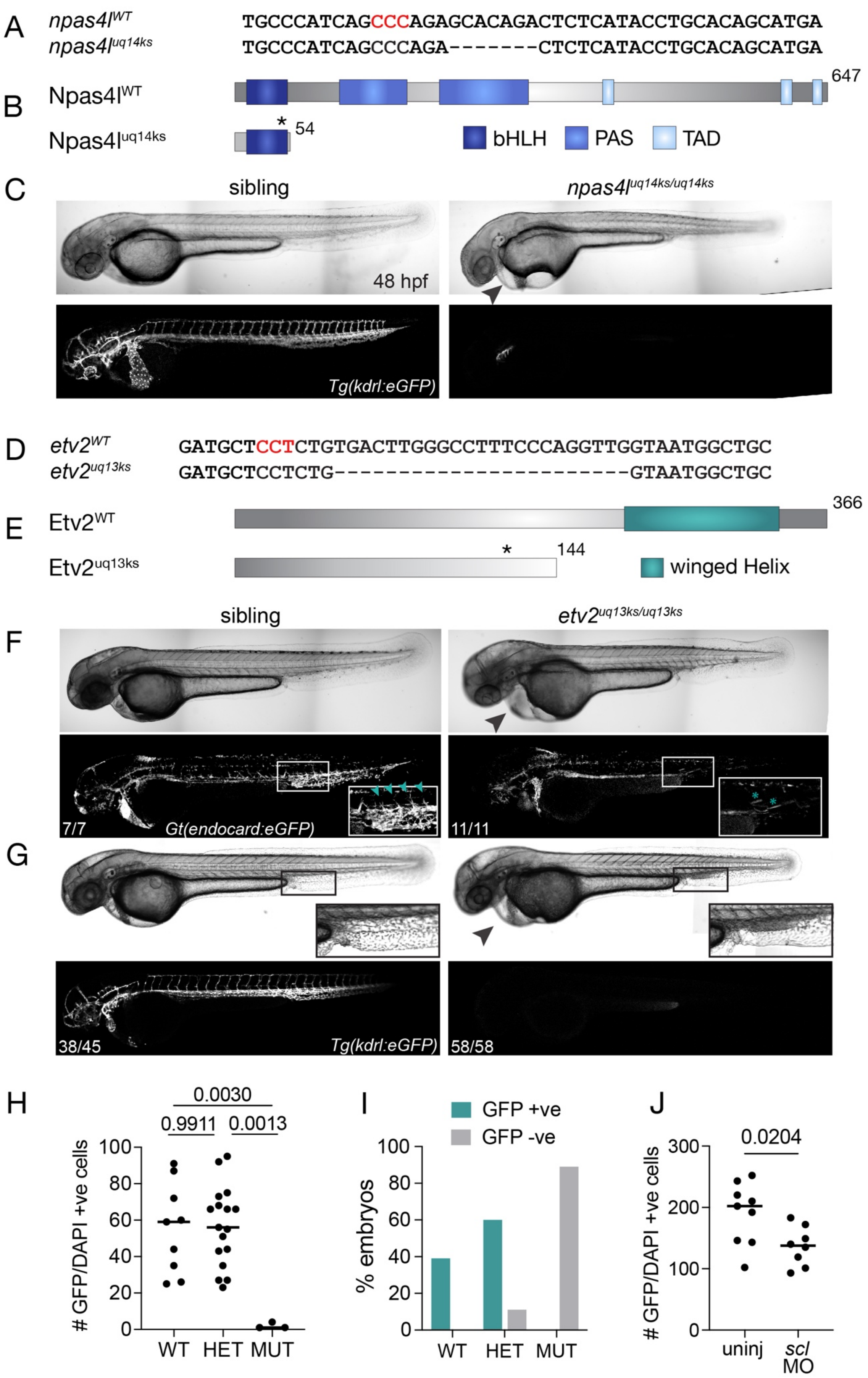

Figure S7

Development • Supplementary information

Fig. S7. A. Sequence of CRISPR-Cas9-induced lesion in *npas4l*^{uq14ks} allele. **B.** Schematic representing the predicted protein resulting from the *npas4l*^{uq14ks} mutation. **C.** Bright field (above) and confocal image of *Tg(kdrl:egfp)* expression (below) in wildtype sibling versus *npas4l*^{uq14ks} mutant. Almost all eGFP expression is absent in homozygous mutants, consistent with previously published alleles. Pronounced pericardial oedema is present in mutant embryos (arrowhead). **D.** Sequence of CRISPR-Cas9-induced lesion in *etv2*^{uq13ks} allele. **E.** Schematic representing the predicted protein resulting from the *etv2*^{uq13ks} mutation. **F.** Bright field (above) and confocal image of *Gt(endocard:egfp)* expression (below) in wildtype sibling versus *etv2*^{uq13ks} mutant. Pronounced pericardial oedema is present in mutant embryos (black arrowhead). The *Gt(endocard:egfp)* reporter shows intersegmental venous sprouts (teal arrowheads) present in siblings that are absent from *etv2*^{uq13ks} mutants (inset), accompanied by reduced endothelial expression and ectopic expression in skeletal muscle fibres, as previously reported (inset; teal asterisks) (Chestnut et al., 2020). **G.** Bright field (above) and confocal image of *Tg(kdrl:egfp)* expression (below) in wildtype sibling versus *etv2*^{uq13ks} mutant. As above, pericardial oedema is present in mutant embryos (arrowhead) and the caudal vein plexus is absent. Inset: caudal vein plexus is observable by bright field contrast imaging, whereas accumulated blood is observed in mutant embryos. The *kdrl:egfp* transgene was never observed in *etv2* $^{uq13ks/uq13ks}$ homozygous mutant embryos (n=58), whereas homozygous wildtype embryos were always observed to segregate with the *kdrl:egfp* transgene (n=15), suggesting *etv2* is genetically linked with the *Tg(kdrl:egfp)* locus. **H.** Quantification of *Gt(endocard:egfp)* positive cells (determined by DAPI counterstain) in *etv2*$^{+/+}$(WT), *etv2*$^{+/uq13ks}$ (HET) and *etv2* $^{uq13ks/uq13ks}$ (MUT) at 14s. **I.** Genotyping of GFP-positive and GFP-negative embryos for the *etv2*^{uq13ks} allele, confirms genetic linkage to the the *Tg(kdrl:egfp)* locus (χ^2 test: $p < 0.0001$). **J.** Quantification of *Gt(endocard:egfp)* positive cells (determined by DAPI counterstain) in uninjected control and *scl* morphant embryos at 14s.

Development • Supplementary information

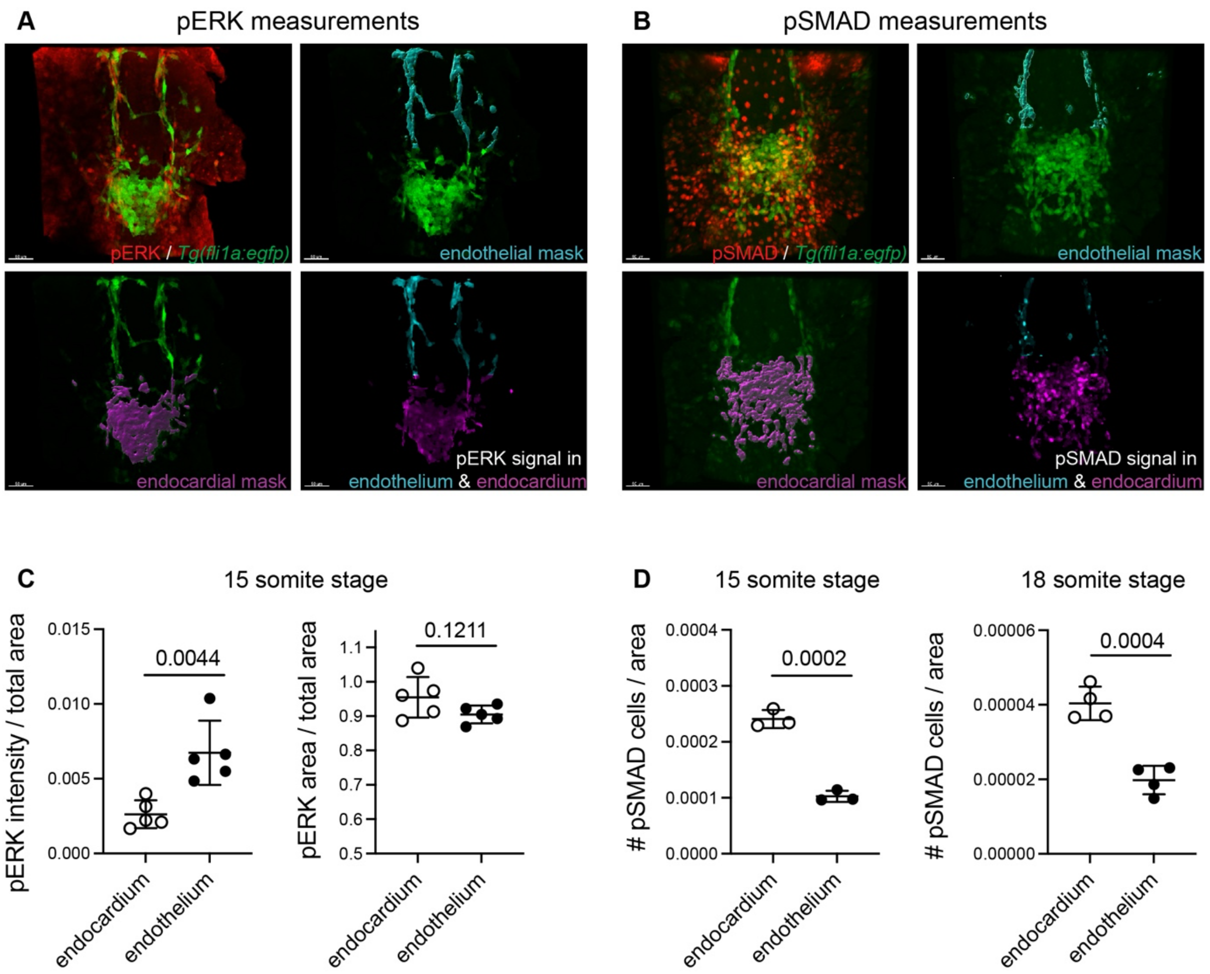

Figure S8

Fig. S8. Representative images of confocal scans for **A.** pERK or **B.** pSMAD and α-GFP immunostaining on *Tg(fli1a:egfp)* background. Images show examples of endocardial or endothelial masks created in Imaris to quantify pERK or pSMAD staining in these two tissues. **C.** and **D.** Dot plots depicting quantification of pERK (**C**) or pSMAD (**D**) in endocardium versus endothelium.

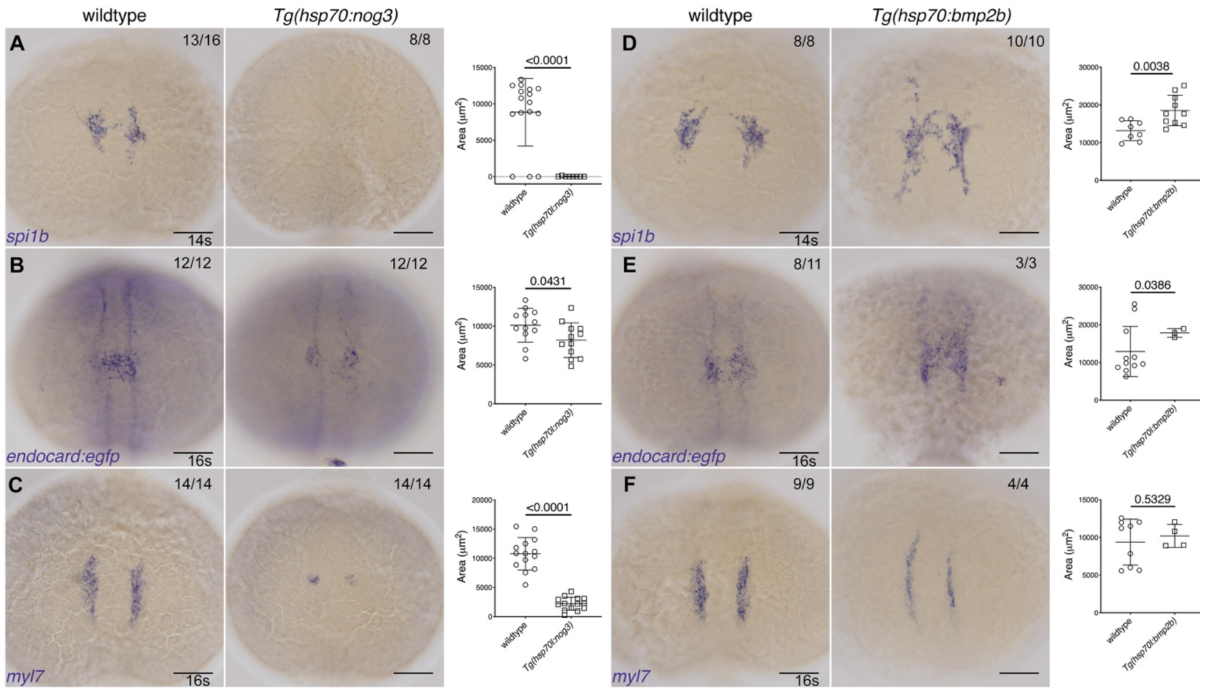

Figure S9

Fig. S9. *In situ* hybridisation for myeloid expression, *spi1b* (**A** and **D**), at the 14s as well as for endocardial, *endocard:egfp* (**B** and **E**), and myocardial, *myl7* (**C** and **F**), expression at the 16s in embryos from crosses of the *Gt(endocard:egfp)* line to either the *Tg(hsp70l:nog3)* or *Tg(hsp70l:bmp2b)* lines. Quantification of the expression is shown in adjacent graphs. Dorsal views are shown with anterior to the top in all images. Scale bars represent 100 µm. P-values are indicated in graphs.

Development • Supplementary information

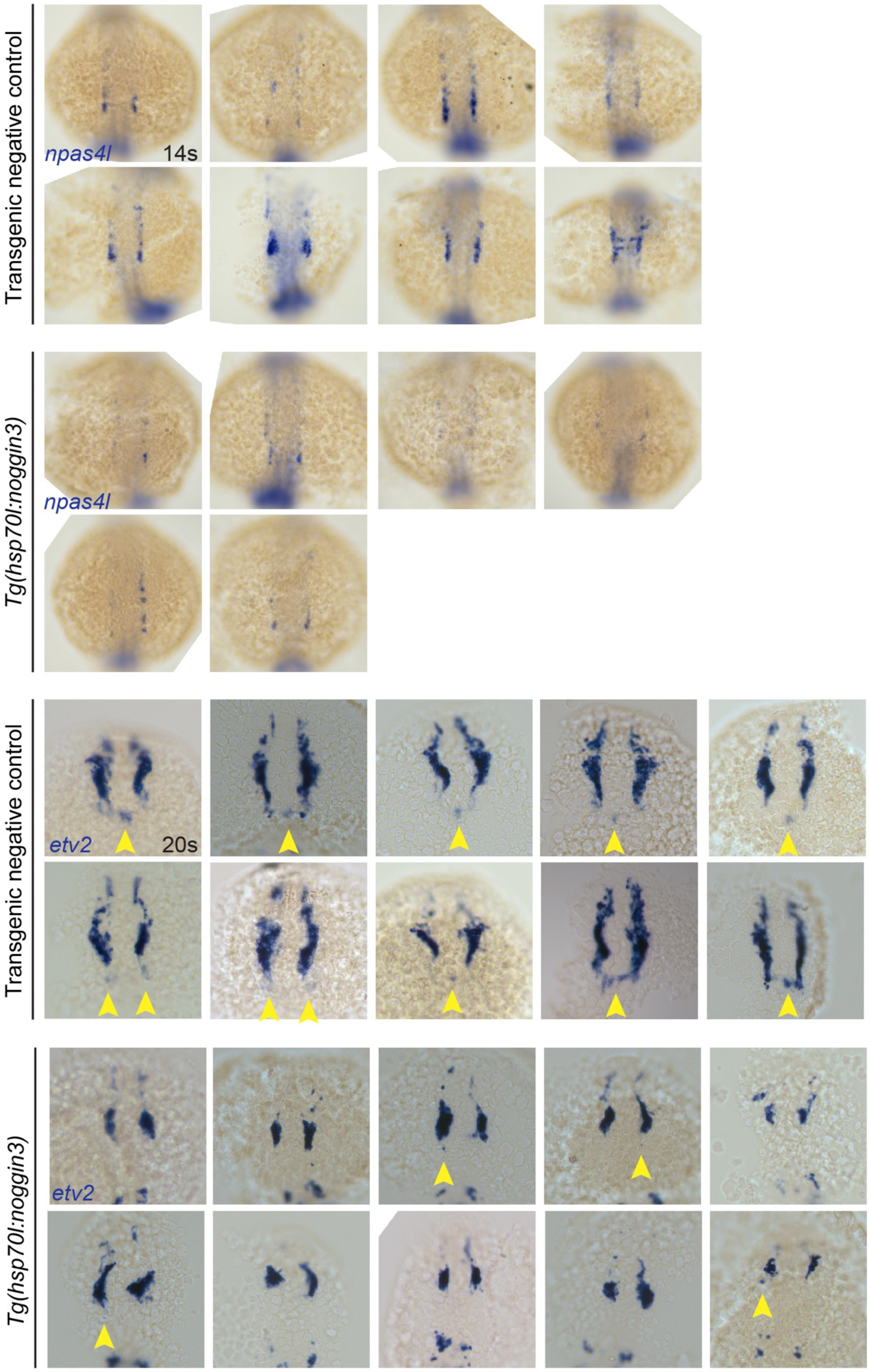

Figure S10

Development • Supplementary information

Fig. S10. *In situ* hybridisation for *npas4l* expression in wildtype sibling controls and *Tg(hsp70l:nog3)* embryos at 14s, showing reduced or absent expression of *npas4l* upon Bmp inhibition. Figure shows one of two experiments performed (total embryos analysed n = 10 for transgenic negative controls and n=8 for transgene positive). *In situ* hybridisation for *etv2* expression in wildtype sibling controls and *Tg(hsp70l:nog3)* embryos at 20s. At 20s, the majority of *etv2* expression is enriched in the presumptive vasculature. A small amount of *etv2* expression can be observed in the developing endocardium (yellow arrowheads). Upon Bmp inhibition, the expression domain is smaller and endocardial expression is greatly reduced (yellow arrowheads). Figure shows one of two experiments (total embryos analysed n = 25 for transgenic negative controls and n=11 for transgene positive)

Development • Supplementary information

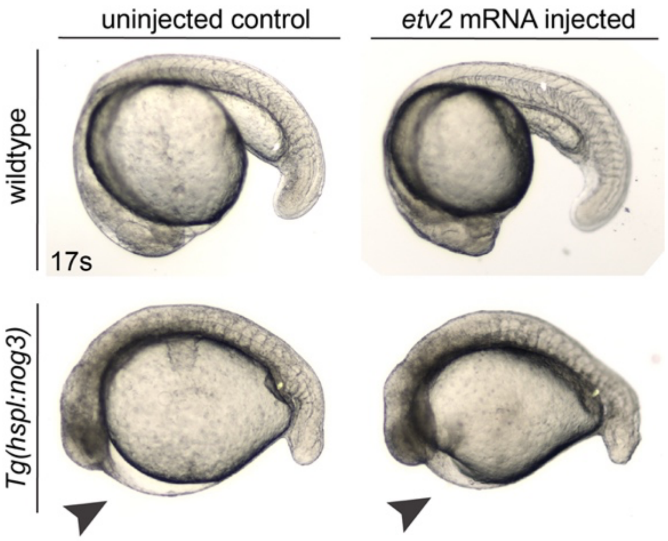

Figure S11

Fig. S11. Bright field lateral view images wildtype of *Tg(hsp70l:nog3)* embryos at 17s, either uninjected or injected with *etv2* mRNA. *Tg(hsp70l:nog3)* embryos heat-shocked at tailbud stage present with a characteristic dorsalisation phenotype. Whilst *etv2* overexpression may rescue the *endocard:egfp* expression in the ALPM, it does not rescue the overall patterning defects of the embryo, nor does it prevent pericardial oedema that forms in *Tg(hsp70l:nog3)* embryos (black arrowhead).

Table S1. Full list of RNAseq analysis and GO terms associated with the differentially expressed data. The lists correspond to the complete list of genes from the differential expression analysis (DE_endocard-egfpVSfliegfp), as well as all significantly up (GO_sig_down_up-list) or down (GO_sig_down_ref-list) gene lists and significantly up or down gene lists with a $\log_2$ Fold Change cut-off of ≥1 (GO_TREAT_up_ref-list) and ≤-1 (GO_TREAT_down_ref-list) applied by TREAT.

Click here to download Table S1

Development • Supplementary information

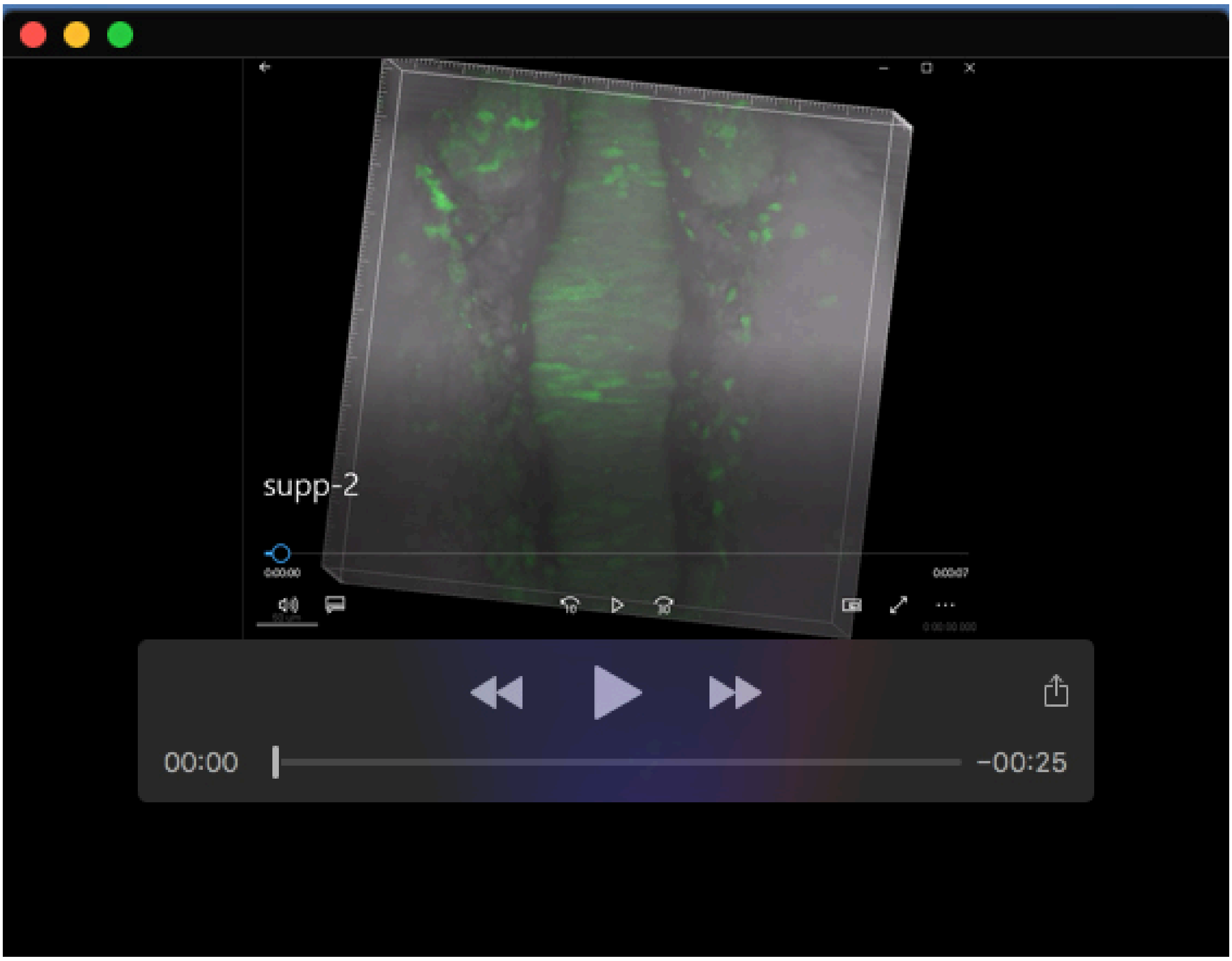

Movie 1.

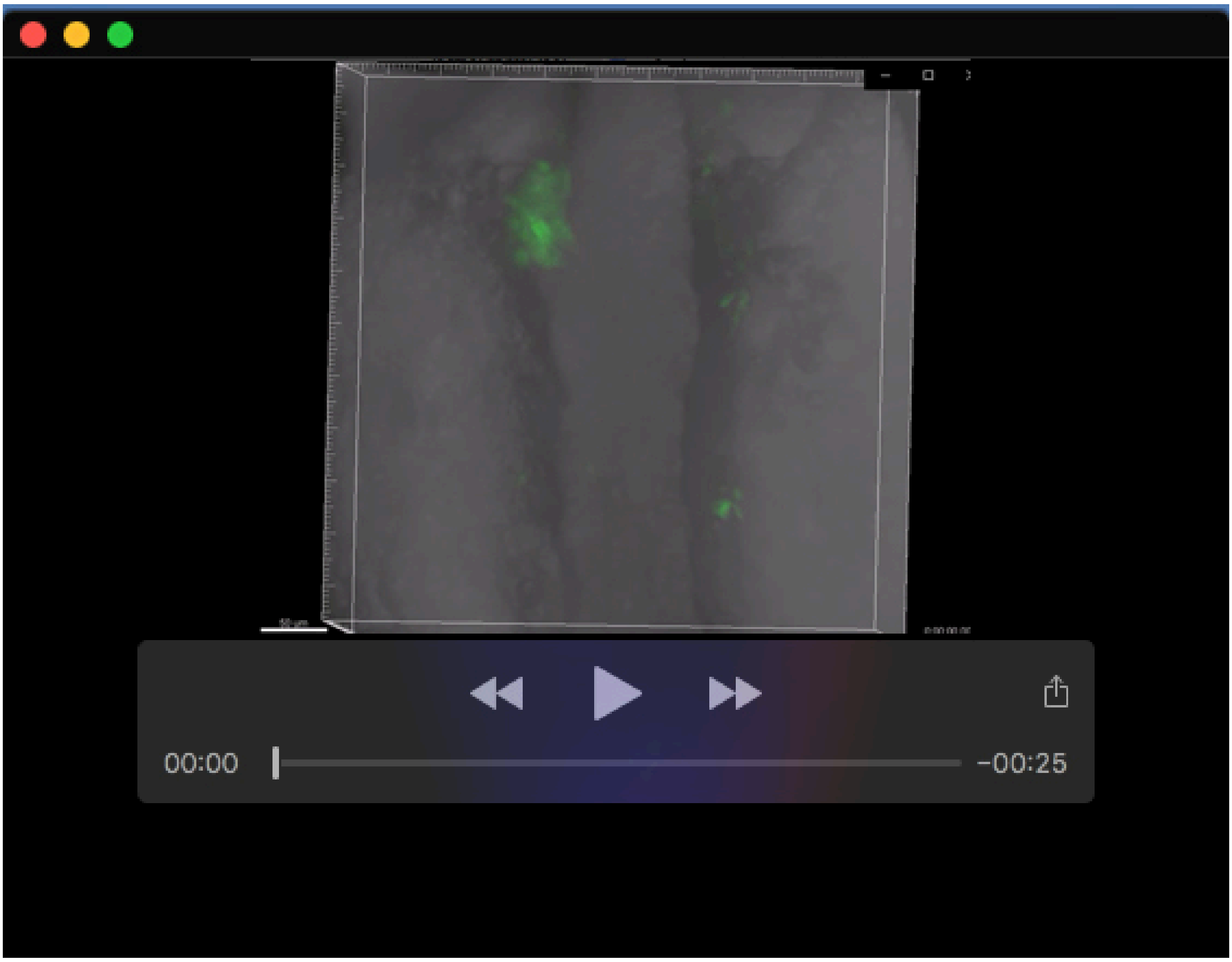

Movie 2.
