# Supplementary material for: Endocardial identity is established during early somitogenesis by Bmp signalling acting upstream of npas4l and etv2
Source: Development. 2022 May 9;149(9):dev190421. doi: 10.1242/dev.190421 (PMC9148566; doi:10.1242/dev.190421)
Supplement: Reviewer comments [file develop-149-190421_review_history.pdf]

Endocardial identity is established during early somitogenesis by Bmp signalling acting upstream of *npas4l* and *etv2*

Samuel J Capon, Veronica Uribe, Nicole Dominado, Ophelia Ehrlich and Kelly Smith DOI: 10.1242/dev.190421

Editor: Benoit Bruneau

Review timeline

Original submission:	10 March 2020
Editorial decision:	14 December 2020
First revision received:	18 February 2022
Editorial decision:	15 March 2022
Second revision received:	27 March 2022
Accepted:	28 March 2022

Original submission

First decision letter

MS ID#: DEVELOP/2020/190421

MS TITLE: Endocardial identity is established during early somitogenesis by Bmp signalling acting upstream of etv2

AUTHORS: Kelly Smith and Samuel Capon

Sincere apologies for the delay in getting back to you. I have now received all the referees reports on the above manuscript, and have reached a decision. The referees' comments are appended below, or you can access them online: please go to BenchPress and click on the 'Manuscripts with Decisions' queue in the Author Area.

The overall evaluation is positive and we would like to publish a revised manuscript in Development, provided that the referees' comments can be satisfactorily addressed, which may include additional experimental data. Please attend to all of the reviewers' comments in your revised manuscript and detail them in your point-by-point response. If you do not agree with any of their criticisms or suggestions explain clearly why this is so.

We are aware that you may currently be unable to access the lab to undertake experimental revisions. If it would be helpful, we encourage you to contact us to discuss your revision in greater detail. Please send us a point-by-point response indicating where you are able to address concerns raised (either experimentally or by changes to the text) and where you will not be able to do so within the normal timeframe of a revision. We will then provide further guidance. Please also note that we are happy to extend revision timeframes as necessary.

Reviewer 1

Advance summary and potential significance to field

The manuscript by Capon and Smith describes identification of a zebrafish endocardial specific gene trap line which is used to study early stages of endocardial specification and differentiation.

© 2022. Published by The Company of Biologists under the terms of the Creative Commons Attribution License (https://creativecommons.org/licenses/by/4.0/).

The authors use this line to identify the transcriptome of endocardial cells and show that these cells are transcriptionally different from vascular endothelial cells at the 8-somite stage, which is much earlier than previously demonstrated. The authors further provide data arguing for BMP and etv2 role in early endocardial specifiction and argue that etv2 functions downstream of BMP signaling in this process.
Overall, the images are of high quality, results are clearly presented and provide a novel insight into the mechanism of endocardial specification. Endocardial gene trap line will be clearly a valuable tool to study endocardial morphogenesis. Identification of endocardial transcriptome is also very interesting and will be a valuable resource for many researchers. Identification of BMP signaling and etv2 role in early endocardial specification is intriguing, although the novely of these observations is somewhat diminished by earlier studies that have implicated these factors in later stages of endocardial differentiation. The mechanism for BMP functioning through etv2 signaling in endocardial morphogenesis is a novel finding, although additional experiments are needed to convincingly demonstrate this outcome.

Comments for the author

Major points:
1. Is there a relative enrichment of pSMAD immunostaining in endocardial cells compared to adjacent cranial vascular endothelial cells? Demonstration of a lower magnification view which would include cranial vasculature or some quantification of staining intensity in both types of cells would help to answer this question.
2. Presented data show that hsp70:bmp2b overexpression induces increased etv2 expression throughout the embryo, in both anterior and posterior sections. This is expected to promote overall vascular endothelial differentiation, including endocardial differentiation. The manuscript currently implies that BMP signals through etv2 to instruct endocardial differentiation, yet the results suggest that BMP overexpression does not specifically affect endocardial lineage and promotes overall endothelial differentiation. This needs to be clearly acknowledged.
3. Etv2 expression does not appear to be reduced in BMP inhibited embryos. This does not support the proposed model that npas4l and etv2 function downstream of BMP signaling to induce endocardial differentiation (npas4l expression was not even analyzed). The data show that BMP overexpression can induce etv2 expression, yet it suggests that BMP signaling is not required for etv2 expression. An alternative model is that BMP and etv2 both provide parallel inputs to promote expression of endocardial genes; in fact previous studies have suggested that BMP signaling and etv2 may directly regulate nfatc1 expression.
4. Rescue experiment of endocard:GFP expression in hsp70l:nog3 embryos injected with etv2 or npas4l mRNA is somewhat puzzling. Because etv2 expression is not reduced in hsp70:nog3 embryos, this argues against the model that etv2 is downstream of BMP. If etv2 was downstream of BMP, then normal level of etv2 present in BMP-inhibited embryos should be sufficient to allow for endocardial differentiation. However, this result could be explained if BMP and etv2 function in parallel. Reduction in the intensity of one input could be compensated by an increased amount of the second signal. In addition, a few controls are needed for this experiment: a) Are the Tg (hsp70:nog3) embryos shown in Fig. 6A and etv2-injected hsp:nog3 embryos shown in Fig. 7B from the same experiment? It is critical to compare and show etv2 RNA-injected and uninjected control hsp70:nog3 embryos that are from the same batch and were processed and analyzed in parallel. Also, the area comparison should be performed between hsp70:nog3 uninjected and hsp70: nog3 etv2-injected as well as control uninjected embryos to show the difference. b) Were homozygous hsp70:nog3 fish used for experiments? If not, then please explain how hsp70:nog3 -positive embryos were identified from transgene negative embryos; c) Based on previous reports, etv2 and npas4l overexpression in wild-type embryos results in dramatic expansion of multiple vascular markers and phenotypic abnormalities. It is somewhat surprising that endocard:GFP expression is relatively unaffected in etv2-overexpressing wild-type embryos. Did etv2 overexpression Authors should show expression of selected other vascular markers (such as kdrl) to show that etv2 and npas4l overexpression produces previously reported phenotypes. d) Please include the numbers how many embryos showed the phenotypes displayed in the figure panels out of the total number of embryos, and how many times each experiment was replicated (this is needed for other figures as well).
5. Can the authors provide images that show overall vascular patterning in npas4l and etv2 mutants to demonstrate that they recapitulate previously described defects?

© 2022. Published by The Company of Biologists under the terms of the Creative Commons Attribution License (https://creativecommons.org/licenses/by/4.0/).

6. The text says that myl7 expression was expanded in etv2 mutants at 16 ss (Fig. 4). However, provided data do not support this statement; there is no significant difference in myl7 area between etv2 mutants and siblings.

Minor points:

1. It is apparent that fli1:GFP expression also labels blood cells, in addition to vascular endothelial cells which is supported by identification of gata1, hbbe3 and hbae5 expression. It would be helpful to clarify this in the text, instead of calling this as 'endothelial transcriptome', it is actually a mixture of more than one cell type, primarily endothelial and blood cells.
2. Please check the reference to Fig. 10 on page 12.
3. In the Discussion on page 15, it says "… we detected an expansion of the myocardial lineage in the absence of myeloid and / or endocardial lineages, but no expansion of these lineages in the absence of myocardium". There is no data in this manuscript regarding myeloid and endocardial lineages in the absence of myocardium.
4. The Discussion on page 15, says that "…like scl, etv2 is dispensable for the initiation of the endocardial program". Yet the data in Fig. 4 shows that initiation of endocardial program is inhibited in etv2 mutants differently from scl phenotype.
5. Discussion on page 15 says that "endocardial progenitors failed to migrate to the midline in etv2 mutants, suggesting a defect in early endocardial differentiation". Such a result would suggest a defect in migration, and not necessarily differentiation.

Reviewer 2

Advance summary and potential significance to field

Capon et al. present new data on how endocardial identity is established during early development. They identified a transgenic line, previously shown as lymphatic reporter, Gt(endocard:egfp), as a line to report early endocardial progenitors from 10ss on. These transgenic fish allowed the authors to compare the transcriptome of endocardial vs. endothelial progenitors and to identify further early endocardium-specific genes, which is of high interest in the field. Moreover, Capon et al. describe the defects on endocardial, hematopoietic and myocardial progenitors that occur in newly generated mutants of npas4l and etv2. Finally, the authors describe a role for BMP-signalling during early endocardial specification by using a transgenic gain- and loss of function approach.

The first part of the manuscript focuses on the specificity of the endocardium and distinguishes endocardial from endothelial gene expression. In the second part of the study, functional studies describe the role of BMP signaling in endocardial progenitor biology. However, more functional studies are required clarify the role of this signaling pathway with respect to the expression of endocardial-specific gene expressions.

Comments for the author

Mayor concerns:

1. The authors state in their abstract that "these results describe the differentiation of the endocardium, distinct from endothelium, during early somitogenesis stages, and show it is regulated by npas4l and etv2 downstream of Bmp signalling." However, it is not obvious how this work describes that the differentiation of the endocardium is regulated distinctly from the endothelium. How was it tested that the endothelium is not equally affected by the functional perturbations described in this study?
2. The authors identify the transgenic line Gt(endocard:egfp) as a reporter for endocardial progenitors. However, some questions regarding the specificity of this marker remain. For instance in Fig.1 (15ss, 20ss), a population of GFP+ cells that are not kdrl:mcherry+ are present (especially in the central rostral region). Is this a common observation? What kind of cells could those be?
A more detailed quantification of GFP+/ mcherry+ vs. GFP+/mcherry- vs. GFP-/mcherry+ cell populations would help to better understand the specificity of this line.
Moreover an additional experiment using Tg(fli1a:nGFP) (or similar) x Gt(endocard:egfp) would complement this study and prevent differences in reporter-gene detection due to time differences in fluorescent protein folding.

© 2022. Published by The Company of Biologists under the terms of the Creative Commons Attribution License (https://creativecommons.org/licenses/by/4.0/).

Can the authors exclude that GFP+ cells in Gt(endocard:egfp) fish represent also other progenitors (myocardial, hematopoietic, …) by using other reporter lines or in situ/ immunostainings?
3. A quantification of GFP+ cells in Gt(endocard:egfp) fish would give a better perception on how the specification in terms of cell numbers is affected in etv2 mutants and tal1-morphants (Fig. 4). Moreover, it would be interesting to see the phenotype of the whole embryo at 24hpf of the mutants (npas4l, etv2), if possible, in combination with an endothelial reporter to confirm that there is consistency with the published phenotype.

4. The authors show the importance of the BMP-pathway for endocardial development, which include an increased expression of GFP in Gt(endocard:egfp) embryos upon BMP-GOF. As the authors point out, the functionality of this line and the reason of GFP expression in endocardial progenitors is not clear. This makes it difficult to actually interpret this observed increase of expression. To better assess the effects of BMP-GOF and LOF, the quantification of GFP+ cells in Gt(endocard:egfp) fish and/ or in combination with an endothelial reporter line would help to understand if specification or the number of endocardial cells is indeed affected. This should be shown in addition to in situ hybridization data against GFP as shown in Fig.7A, B.
Moreover, it is not clear in what respect the observed effects of disturbed BMP-signalling are specific to the endocardium and or include defective development of other tissues. Is the general development of the ALPM affected or of other endothelial and hematopoietic populations, upon BMP-GOF and LOF? And what is the consequence on the morphology and function of the heart at 24/ 48 hpf? How does BMP-GOF and LOF affects npas4l-expression?
Can potential changes in heart morphology and vascular development be rescued as well by etv2-mRNA-epression?
5. One experiment that is critically missing is the BMP-GOF effect in npas4l mutants. This is an essential experiment also considering one of the authors main conclusions (lines 428-430): “Together, these results suggest that Bmp signalling and npas4l converge on the activation of etv2 expression to regulate endocardial development.” To make this statement, the authors would need to show whether the overexpression of Bmp2b in npas4l mutants indeed activates etv2 expression (which would be expected in case of parallel pathways converging on etv2).
6. Line 398-401. In their discussion the authors refer to very interesting data: “Surprisingly,examining etv2 mutants at 48 hpf revealed the presence of an endocardial layer and endocard:egfp expression, indicating that the endocardium was still able to form, although on rare occasions we did note mutants lacking large segments of endocardium (data not shown).” It would be important to show this data.

Minor concerns:
1.Lines 85-89: The authors state that “In agreement with this model, the zebrafish cloche mutant lacks all endocardium, yet retains some endothelium, suggesting that the endocardium and vascular endothelium have distinct developmental origins (Stainier et al.,1995).” However, since cloche/npas4l is not specific to the endocardium but also deletes much of the vasculature, it rather appears that endocardium and some endothelial lineages share a common origin as defined by the activity of Npas4l.
2.Line 51: Expression of nfatc1 is not endocardium-specific.
3.The representation of Fig. 7B does not seem to be appropriate. The listing of all conditions, including controls, in this figure would help the reader to better perceive the defects that occur upon BMP-LOF or not and if those are rescued. This means, images and quantifications of data should be shown from the following conditions in one panel: wildtype + etv2mRNA, wildtype + control (or no) mRNA, Tg(hsp70l:nog3) + etv2mRNA, Tg(hsp70l:nog3) + control (or no) mRNA. Accordingly, this should be shown for npas4l-mRNA injection experiments.
4.It would be interesting, if the authors could comment and maybe show potential BMP-pathway elements that fulfil this described role of BMP signalling. Does the endocardial transcriptome data reveal any candidates?
5.Line 306. Here the authors mention Fig. 10 which is not present in the manuscript.
6.Could the authors comment on the reason for FACS sorting only Gt(endocard:egfp) high expressing cells for the RNA-seq analyses? Was this done to separate endocardium versus myocardium? Does the GO term analysis which highlights myocardial functional terms point at the fact that this marker transgene is not specific to the endocardium?
7.Line 359: What is meant with apical regulators?
8. Line 328-330. Here, the authors claim that “These results show that the endocardium is specified earlier than previously appreciated in zebrafish development...” However, this

© 2022. Published by The Company of Biologists under the terms of the Creative Commons Attribution License (https://creativecommons.org/licenses/by/4.0/).

manuscript does not provide any functional proof that cells with an expression of Gt(endocard:egfp) are indeed specified already as endocardial cells at these early stages. This paper at best suggests that the expression domain of this transgenic marker more or less fits what would be expected of a fate map for endocardium at these somitogenesis stages.

First revision

Author response to reviewers' comments

Reviewer 1

Summary:
The manuscript by Capon and Smith describes identification of a zebrafish endocardial specific gene trap line which is used to study early stages of endocardial specification and differentiation. The authors use this line to identify the transcriptome of endocardial cells and show that these cells are transcriptionally different from vascular endothelial cells at the 8- somite stage, which is much earlier than previously demonstrated. The authors further provide data arguing for BMP and etv2 role in early endocardial specifiction and argue that etv2 functions downstream of BMP signaling in this process.

Overall, the images are of high quality, results are clearly presented and provide a novel insight into the mechanism of endocardial specification. Endocardial gene trap line will be clearly a valuable tool to study endocardial morphogenesis. Identification of endocardial transcriptome is also very interesting and will be a valuable resource for many researchers. Identification of BMP signaling and etv2 role in early endocardial specification is intriguing, although the novely of these observations is somewhat diminished by earlier studies that have implicated these factors in later stages of endocardial differentiation. The mechanism for BMP functioning through etv2 signaling in endocardial morphogenesis is a novel finding, although additional experiments are needed to convincingly demonstrate this outcome.

Major points:

POINT 1:
Is there a relative enrichment of pSMAD immunostaining in endocardial cells compared to adjacent cranial vascular endothelial cells? Demonstration of a lower magnification view which would include cranial vasculature or some quantification of staining intensity in both types of cells would help to answer this question.

RESPONSE:
Yes, there is. To confirm this, we have analysed immunofluorescence data using quantitative methods. We counted (using automation) the number of pSMAD-positive cells in the endocardium versus the adjacent endothelium and normalised cell counts to the area measured. We found significantly more pSMAD-positive cells in the endocardium compared with the endothelium. To verify this was not an effect of the region, we quantified pERK signal in endocardium versus endothelium. Because pERK is not nuclear but cytoplasmic, we measured the number of voxels (area) with a pERK signal as well as the fluorescence intensity of the pERK signal within endocardium and endothelial regions. Unlike pSMAD, we observed no difference between endocardium and endothelium for the area positive for pERK activity and we observed significantly higher fluorescence intensity in the endothelium versus endocardium. Together, these data suggest that Bmp signalling is significantly and selectively higher within endocardial cells compared with adjacent endothelium. We include this new analysis in existing Figure 5 as well as a new figure (Fig S8) and associated text on page 9-10 of the manuscript.

POINT 2:
Presented data show that hsp70:bmp2b overexpression induces increased etv2 expression throughout the embryo, in both anterior and posterior sections. This is expected to promote overall vascular endothelial differentiation, including endocardial differentiation. The manuscript

© 2022. Published by The Company of Biologists under the terms of the Creative Commons Attribution License (https://creativecommons.org/licenses/by/4.0/).

currently implies that BMP signals through etv2 to instruct endocardial differentiation, yet the results suggest that BMP overexpression does not specifically affect endocardial lineage and promotes overall endothelial differentiation. This needs to be clearly acknowledged.

RESPONSE:
We agree with the reviewer. We suggest that Bmp signalling is more active in endocardial tissue compared with adjacent endothelium (see analysis for point 1 above), at least for this timepoint. However, this data does not assess whether the endocardium is more sensitive to Bmp signalling nor does it negate a role for Bmp signalling other endothelial tissues. We have amended discussion around this on page 13.

POINT 3a:
Etv2 expression does not appear to be reduced in BMP inhibited embryos. This does not support the proposed model that npas4l and etv2 function downstream of BMP signaling to induce endocardial differentiation (npas4l expression was not even analyzed).

RESPONSE:
We agree that our evidence for an effect of Bmp inhibition on *etv2* expression levels was weak. This was complicated by a lack of *etv2* expression observed in the endocardium at the timepoint examined (to be clear, we believe expression is present at 15s and our RNAseq data supports that however we are unable to detect it by ISH). To address this, we repeated our analysis of *etv2* expression at 15s and at an additional timepoint (20s) and stained for longer. We observed no detectable expression in endocardium at the 15s (the timepoint originally analysed) but did see it at 20s. At 20s, we observe a reduction in the *etv2* expression domain in the ALPM, both in presumptive cranial vasculature and in endocardium upon Bmp inhibition. In fact, the small amount of *etv2* expression detected in the endocardium (yellow arrowheads) was absent in the majority of Bmp inhibited embryos. We include this data in a new supplementary figure (Fig S10).

To address the absence of *npas4l* analysis, we examined whether *npas4l* expression is also affected by Bmp inhibition. *npas4l* expression rapidly diminishes after 15s (Reischauer et al., 2016 - PMID: 27411634). As such, we investigated the effect of Bmp inhibition on *npas4l* expression at 14s. Whilst expression was weak at this stage and not abolished in Bmp inhibited embryos, we observed a clear and obvious reduction in *npas4l* expression in *Tg(hsp70l:nog3)* embryos, compared with transgene-negative sibling controls. This new data has been included in supplementary Figure S10 and accompanying text of the manuscript (page 10 & 11). Together, these data support a role for *npas4l* and *etv2* functioning downstream of Bmp signalling in endothelium.

POINT 3b:
The data show that BMP overexpression can induce etv2 expression, yet it suggests that BMP signaling is not required for etv2 expression. An alternative model is that BMP and etv2 both provide parallel inputs to promote expression of endocardial genes; in fact, previous studies have suggested that BMP signaling and etv2 may directly regulate nfatc1 expression.

RESPONSE:
The above data and discussion notwithstanding, we agree that this is also possible. Bmp plays multiple successive roles in many stages of cardiac development, often confounding interpretation of its role in specific processes. We believe it is entirely plausible that it plays a role both in the induction of endothelial fate, by interacting with key drivers of endocardial fate, such as *etv2*, and downstream of *etv2* in the endothelium. The complexity of this regulatory loop is now elaborated on page 13 of the discussion. In addition, we provide stronger evidence here for the role Bmp signalling plays in inducing both *npas4l* and *etv2* expression.

POINT 4a:
Rescue experiment of endocard:GFP expression in hsp70l:nog3 embryos injected with etv2 or npas4l mRNA is somewhat puzzling. Because etv2 expression is not reduced in hsp70:nog3 embryos, this argues against the model that etv2 is downstream of BMP. If etv2 was downstream of BMP, then normal level of etv2 present in BMP-inhibited embryos should be sufficient to allow for endocardial differentiation. However, this result could be explained if BMP and etv2 function in parallel. Reduction in the intensity of one input could be compensated by an increased amount of the second signal.

© 2022. Published by The Company of Biologists under the terms of the Creative Commons Attribution License (https://creativecommons.org/licenses/by/4.0/).

RESPONSE:
We have provided additional evidence of Bmp signalling acting upstream of both *npas4l* and *etv2*. We believe this new data clarifies why the rescue of endocardial GFP expression is achievable by *etv2* and *npas4l* mRNA injection.

POINT 4b:
In addition, a few controls are needed for this experiment: a) Are the Tg (hsp70:nog3) embryos shown in Fig. 6A and etv2-injected hsp:nog3 embryos shown in Fig. 7B from the same experiment? It is critical to compare and show etv2 RNA-injected and uninjected control hsp70:nog3 embryos that are from the same batch and were processed and analyzed in parallel.

RESPONSE:
We agree and, no, these animals were not from the same experiment. To correct this short-coming, we have repeated the experiment twice, with all uninjected and injected controls stained at the same time. Post-staining, embryos were imaged, followed by genotyping for the heat-shock promoter, providing a blinded experiment. We have performed this for both *etv2* and *npas4l* mRNA and we observe the same outcome: that is, *egfp* expression is reduced or absent upon *hs:noggin* induction, and expression is restored in *hs:noggin* embryos injected with either *etv2* or *npas4l* mRNA. We have now replaced the previous data with the revised data that includes all control categories in Fig 7.

POINT 4c:
Also, the area comparison should be performed between hsp70:nog3 uninjected and hsp70: nog3 etv2-injected as well as control uninjected embryos to show the difference.

RESPONSE:
This quantification has now been performed and is included in Fig 7 along with the new images.

POINT 4d:
1. Were homozygous hsp70:nog3 fish used for experiments? If not, then please explain how hsp70:nog3 -positive embryos were identified from transgene negative embryos;

RESPONSE:
We maintain the *Tg(hsp70l:nog3)* (and *Tg(hs:bmp)*) line in a heterozygous state, generating new generations by outcrossing to wildtype strains, genotyping adults and culling off transgene-negative fish. This extends to experimentation, where we outcross *Tg(hsp70l:nog3)* fish to the transgenic reporter. This permits 50% of all clutches to contain transgenic-negative controls within the same clutch. For genotyping, all phenotyping is performed first (such as, in situ and imaging), followed by genotyping by PCR for the heat-shock transgene. This ensures experiments with this line are always controlled and staged within the experiment and are performed blind. To make this approach clear, we have now included additional explanation within the methods (page 19-21).

POINT 4e:
2. Based on previous reports, etv2 and npas4l overexpression in wild-type embryos results in dramatic expansion of multiple vascular markers and phenotypic abnormalities. It is somewhat surprising that endocard:GFP expression is relatively unaffected in etv2- overexpressing wild-type embryos. Did etv2 overexpression Authors should show expression of selected other vascular markers (such as kdrl) to show that etv2 and npas4l overexpression produces previously reported phenotypes.

RESPONSE:
We have now repeated the experiment and we don't observe expansion in *egfp* staining upon *etv2* overexpression (Figure 7). Compared with published observations, we injected slightly lower amounts of *etv2* mRNA concentration (30pg in our study versus 35pg in PMID:32628937, for example). This minor difference in concentration may explain why an increase was not observed or perhaps a difference in injection bolus size used between laboratories. Alternatively, the endocardium may be more tightly regulated than the vasculature, explaining why a drastic expansion of endocardium is not observed upon *etv2* overexpression, than if visualising with vascular markers. Irrespective of this, we have repeated these injections and stainings at two different locations (in Brisbane and in Melbourne, due to laboratory relocation mid-revision), and

© 2022. Published by The Company of Biologists under the terms of the Creative Commons Attribution License (https://creativecommons.org/licenses/by/4.0/).

by two different researchers therefore we are confident that what we observe is reproducible in our hands.

Like *etv2*, no significant difference in *egfp* staining area was observed for *npas4l* overexpression, although there was higher variability for *npas4l* overexpression observed (see Figure 7F - quantification). This observation may come down to levels: the amount injected was considerably lower than previously reported (10pg in our study versus 25 and 50pg in PMID: 31097478 and PMID: 27411634). This lack of expansion of endocardium is consistent with what we observe for *etv2* overexpression. The revised data is now included in Figure 7.

POINT 4f:
3. Please include the numbers how many embryos showed the phenotypes displayed in the figure panels out of the total number of embryos, and how many times each experiment was replicated (this is needed for other figures as well).

RESPONSE:
The number of embryos examined is now included in each image and the number of experimental replicates added to the methods (page 18).

POINT 5:
Can the authors provide images that show overall vascular patterning in *npas4l* and *etv2* mutants to demonstrate that they recapitulate previously described defects?

RESPONSE:
We now include lateral view images of both the *etv2* and *npas4l* mutants generated and used in this study. As shown by brightfield, we observe pericardial oedema in both mutants and we also observe blood accumulation in the caudal plexus of *etv2* mutants (inset), consistent with previously published mutant alleles. To image the vasculature, we crossed these alleles onto the *Tg(kdrl:eGFP)* reporter and performed confocal imaging. For *cloche/npas4l* mutants, we observed the characteristic fluorescence of the pharyngeal arch endoderm only in mutant embryos (as reported in PMID: 27411634). For *etv2*, however, we never observed GFP expression. To determine whether this was due to diminished fluorescence (as reported in PMID:17125752), we genotyped for both *etv2* and *egfp*. Surprisingly, we never found $etv2^{uq13ks/uq13ks}$ co-segregating with *egfp* and, reciprocally, we observed $etv2^{wt/wt}$ embryos were always *egfp*-positive. To validate this statistically, we performed a Chi-squared test and confirm that the *etv2* locus and the insertion site for the *kdrl:egfp* transgene in the *Tg(kdrl:egfp)* transgenic line are genetically linked, a detail that has not reported (at least, not to our knowledge). We have included this new information in Figure S7.

POINT 6:
The text says that myl7 expression was expanded in etv2 mutants at 16 ss (Fig. 4). However, provided data do not support this statement; there is no significant difference in myl7 area between etv2 mutants and siblings.

RESPONSE:
Whilst the *myl7* expression domain hasn't increased in area, the length of the expression domains is expanded in the anterior-posterior (A-P) direction (presumably due to endothelial progenitors ectopically expressing *myl7*, as has previously been reported for *tal1* and *cloche* loss-of-function conditions: PMID:17681136). To confirm this, we measured the length of the A-P expression domain in these different loss-of-function contexts, including *etv2*, and observe expansion of *myl7* in all loss-of-function scenarios. We add the new data to existing Figure 4.

MINOR POINTS:
1. It is apparent that fli1:GFP expression also labels blood cells, in addition to vascular endothelial cells, which is supported by identification of gata1, hbbe3 and hbae5 expression. It would be helpful to clarify this in the text, instead of calling this as 'endothelial transcriptome', it is actually a mixture of more than one cell type, primarily endothelial and blood cells.

© 2022. Published by The Company of Biologists under the terms of the Creative Commons Attribution License (https://creativecommons.org/licenses/by/4.0/).

This has now been amended on page 6.

2. Please check the reference to Fig. 10 on page 12.

This has now been amended

3. In the Discussion on page 15, it says "… we detected an expansion of the myocardial lineage in the absence of myeloid and / or endocardial lineages, but no expansion of these lineages in the absence of myocardium". There is no data in this manuscript regarding myeloid and endocardial lineages in the absence of myocardium.

This has now been removed from the manuscript.

4. The Discussion on page 15, says that "…like scl, etv2 is dispensable for the initiation of the endocardial program". Yet the data in Fig. 4 shows that initiation of endocardial program is inhibited in etv2 mutants, differently from scl phenotype.

This has now been amended

5. Discussion on page 15 says that "endocardial progenitors failed to migrate to the midline in etv2 mutants, suggesting a defect in early endocardial differentiation". Such a result would suggest a defect in migration, and not necessarily differentiation.

This has now been amended

Reviewer 2

Summary:
Capon et al. present new data on how endocardial identity is established during early development. They identified a transgenic line, previously shown as lymphatic reporter, Gt(endocard:egfp), as a line to report early endocardial progenitors from 10ss on. These transgenic fish allowed the authors to compare the transcriptome of endocardial vs. endothelial progenitors and to identify further early endocardium-specific genes, which is of high interest in the field. Moreover, Capon et al. describe the defects on endocardial, hematopoietic and myocardial progenitors that occur in newly generated mutants of npas4l and etv2. Finally, the authors describe a role for BMP-signalling during early endocardial specification by using a transgenic gain- and loss of function approach.

The first part of the manuscript focuses on the specificity of the endocardium and distinguishes endocardial from endothelial gene expression. In the second part of the study, functional studies describe the role of BMP signaling in endocardial progenitor biology.
However, more functional studies are required clarify the role of this signaling pathway with respect to the expression of endocardial-specific gene expressions.

Mayor concerns:

POINT 1:
The authors state in their abstract that "these results describe the differentiation of the endocardium, distinct from endothelium, during early somitogenesis stages, and show it is regulated by npas4l and etv2 downstream of Bmp signalling." However, it is not obvious how this work describes that the differentiation of the endocardium is regulated distinctly from the endothelium. How was it tested that the endothelium is not equally affected by the functional perturbations described in this study?

RESPONSE:
This is fair criticism. We have not tested functional regulation relative to the vascular endothelium. We have shown that fli1a:eGFP-positive cells versus endocard:GFP-positive cell are transcriptionally different. Furthermore, we have confirmed by in situ staining that some of the differentially expressed genes (markers *gfp, nfatc1, cbfa2t3* and others in Fig S3*)* are enriched in the endocardium. We now also include additional analysis that shows signalling within the two

© 2022. Published by The Company of Biologists under the terms of the Creative Commons Attribution License (https://creativecommons.org/licenses/by/4.0/).

tissues is different (more intense pERK1/2 in vasculature, compared with endocardium and broader and more intense staining for pSmad1/5/8 in endocardium than in vasculature). Despite these data, we take the reviewers point and, as such, have toned down our language around some of these claims in favour of alluding to the transcriptional and signalling differences between the two tissues.

POINT 2a:
The authors identify the transgenic line Gt(endocard:egfp) as a reporter for endocardial progenitors. However, some questions regarding the specificity of this marker remain. For instance in Fig.1 (15ss, 20ss), a population of GFP+ cells that are not kdrl:mcherry+ are present (especially in the central rostral region). Is this a common observation? What kind of cells could those be?

RESPONSE:
It is not a common observation to observe endocardial:GFP+ / kdrl:mCherry- cell specifically in the central rostral region. In this revision, we have added multiple data that include images of the Gt(endocard:gal4) line overlaid with other endothelial reporters (*kdrl:gfp* and *fli:gfp*) providing examples of this (see FigS1 and S6). In addition, we do observe Gt(endocard:egfp) expression in at least presumptive blood cells (visible from the migratory behaviour observed in some cells during timelapse imaging; Movie 1) as well as weak expression in myocardium (Fig S2).
To clarify and extend this, we have performed additional expression analysis by comparing the expression domains of myeloid, myocardium, endocardium and vasculature in relation to one another. We performed double-fluorescent ISH at 15s and observe close to mutually exclusive expression patterns and we performed immunostaining and imaged double transgenics (Gt(endocard:rfp) overlaid with kdrl:egfp) at 20s showing the endocardium is a subset of endothelium. We have added this data as a new Supplementary Figure (Figure S6) and include description on this point on page 8.

POINT 2b:
A more detailed quantification of GFP+/ mcherry+ vs. GFP+/mcherry- vs. GFP-/mcherry+ cell populations would help to better understand the specificity of this line. Moreover an additional experiment using Tg(fli1a:nGFP) (or similar) x Gt(endocard:egfp) would complement this study and prevent differences in reporter-gene detection due to time differences in fluorescent protein folding. Can the authors exclude that GFP+ cells in Gt(endocard:egfp) fish represent also other progenitors (myocardial, hematopoietic, …) by using other reporter lines or in situ/ immunostainings?

RESPONSE:
This is an excellent suggestion and we have completed several experiments to address this. Firstly, we have performed double ISH analysis for expression of endocardium (gfp), myocardium (myl7) and myeloid (spi1b/pu.1) to examine co-localisation or mutual exclusivity in expression (Fig S6 and page 8). We find little-to-no overlap in expression. In addition, we outcrossed *Gt(SAGFF27C)* to a Tg(4xuas:RFP) line (bred to remove the Tg(4xuas:egfp)) and then crossed this to the Tg(kdrl:egfp) line to perform reciprocal comparison of expression to what was done in Fig 1. Unfortunately, due to the difference in fluorescent protein maturation, we were unable to observe RFP expression driven by *Gt(SAGFF27C)* until 20s. At this timepoint, we observed a high percentage of overlap between most *Gt(SAGFF27C)/ Tg(4xuas:RFP)* expressing cells with *Tg(kdrl:egfp)*-positive cells (mean = 93%). This suggests that the GFP+/Mcherry- cells observed in Fig 1 may be due to patchy expression of the *Tg(kdrl:mCherry)* reporter. We have now included this new data in Fig S1 with accompanying text on page 5.

POINT 3:
A quantification of GFP+ cells in Gt(endocard:egfp) fish would give a better perception on how the specification in terms of cell numbers is affected in etv2 mutants and tal1-morphants (Fig. 4). Moreover, it would be interesting to see the phenotype of the whole embryo at 24hpf of the mutants (npas4l, etv2), if possible, in combination with an endothelial reporter to confirm that there is consistency with the published phenotype.

RESPONSE:
We have now performed cell counts on *etv2* mutants and *tal1* morphants for the Gt(endocard:egfp) line. We find that both mutants and morphants have significantly fewer endocardial cells than

© 2022. Published by The Company of Biologists under the terms of the Creative Commons Attribution License (https://creativecommons.org/licenses/by/4.0/).

sibling controls, although the effect is subtle (~25% reduction) in *tal1* morphants, whereas there is almost a complete loss (98% reduction) in *etv2* mutants compared with sibling controls. We now include this data in Fig S7 and accompanying text on page 9. Additionally, we now include images of both the *etv2* and *npas4l* mutants generated and used in this study. This is included in a revised Fig S7 and accompanying text on page 8, validating the phenotypes of the models used in this study. This figure shows lateral view brightfield images as well as the vasculature, from crossing these mutants to the *Tg(kdrl:GFP)* background. As described above, the observed phenotypes are consistent with previously published alleles (PMID: 27411634 and PMID:17125752).

POINT 4a:
The authors show the importance of the BMP-pathway for endocardial development, which include an increased expression of GFP in Gt(endocard:egfp) embryos upon BMP-GOF. As the authors point out, the functionality of this line and the reason of GFP expression in endocardial progenitors is not clear. This makes it difficult to actually interpret this observed increase of expression. To better assess the effects of BMP-GOF and LOF, the quantification of GFP+ cells in Gt(endocard:egfp) fish and/ or in combination with an endothelial reporter line would help to understand if specification or the number of endocardial cells is indeed affected. This should be shown in addition to in situ hybridization data against GFP as shown in Fig.7A, B.

RESPONSE:
We have performed cell counts for both BMP-GOF and LOF and include the new data in Figure 6 as well as revised text on page 10. This new data confirms what was observed for the BMP-LOF, i.e. reduced staining area and decreased cell number for endocardial cells. Interestingly, we do not observe a statistically significant increase in cell number upon BMP- GOF. This contrasts with what we observe for the staining area and validates the concerns of the reviewer. We have now rewritten sections of the manuscript that claim Bmp signalling can expand the endocardial expression domain and remove mention of its functioning with sufficiency.

POINT 4b:
Moreover, it is not clear in what respect the observed effects of disturbed BMP-signalling are specific to the endocardium and or include defective development of other tissues. Is the general development of the ALPM affected or of other endothelial and hematopoietic populations, upon BMP-GOF and LOF?

RESPONSE:
Previously published studies (PMID: 16527746 and 22247485) have described a role for Bmp signalling in hematopoiesis and development of the myocardium. In PMID: 16527746, the authors also report that gain- or loss-of-function for Bmp signalling had no effect on endothelial populations (as determined by *scl* and *fli1* staining). To reflect this and place our work in that context, we have now included additional description of these previous studies in the introduction on pages 4 of the manuscript.

POINT 4c:
And what is the consequence on the morphology and function of the heart at 24/ 48 hpf?

RESPONSE:
Embryos that undergo BMP-GOF and LOF as described in this manuscript have morphological defects on their overall axial patterning (as reported previously PMID: 9409664) as well as cardiac defects (via effects on the myocardium [PMID: 17395172 and 18267096]). These phenotypes prevent development through to 24 and 48 hpf. Nonetheless, to demonstrate the gross morphological effect of Bmp inhibition and whether *etv2* mRNA overexpression can rescue this (it cannot), we now include a new supplementary figure (Fig S11) showing the overall phenotype of the embryos at 17s.

POINT 4d:
How does BMP-GOF and LOF affects npas4l-expression?

RESPONSE:
We have now performed ISH analysis for *npas4l* in LOF embryos. These data show that, like *etv2*, *npas4l* expression is decreased upon BMP-LOF. This new data is now included in Fig S10

© 2022. Published by The Company of Biologists under the terms of the Creative Commons Attribution License (https://creativecommons.org/licenses/by/4.0/).

POINT 4e:
Can potential changes in heart morphology and vascular development be rescued as well by etv2-mRNA-epression?

As described above, the additional axial patterning phenotypes observed upon BMP-GOF and LOF complicate analysis of later stages to examine heart morphology. Because of the previously described role in cardiac laterality and myocardial morphogenesis (PMID: 17395172 and 18267096), heart morphology is considerably compromised, irrespective of the health and development of the endocardium. To demonstrate this, we have taken lateral view brightfield images of 17s embryos that are either wildtype (negative for transgene) or *Tg(hsp70l:nog3)*, with and without *etv2* mRNA injection. It is clear from these images that the axial patterning defects are not rescued by *etv2* injection and that pericardial oedema remains, suggesting that heart morphology is still disrupted. We include this data as a new Supplementary Figure (Fig S11).

POINT 5:
One experiment that is critically missing is the BMP-GOF effect in npas4l mutants. This is an essential experiment also considering one of the authors main conclusions (lines 428-430): “Together, these results suggest that Bmp signalling and npas4l converge on the activation of etv2 expression to regulate endocardial development.” To make this statement, the authors would need to show whether the overexpression of Bmp2b in npas4l mutants indeed activates etv2 expression (which would be expected in case of parallel pathways converging on etv2).

RESPONSE:
We agree with the reviewer and accordingly have completed this experiment. As described above, *npas4l* expression is decreased upon BMP-LOF, consistent with what is observed for *etv2* (which is an immediate downstream target of *npas4l*; PMID: 27411634). This data is now included in Fig S10 and strengthens the evidence for a role for Bmp signalling upstream of *etv2*.

POINT 6:
Line 398-401. In their discussion the authors refer to very interesting data: “Surprisingly,examining etv2 mutants at 48 hpf revealed the presence of an endocardial layer and endocard:egfp expression, indicating that the endocardium was still able to form, although on rare occasions we did note mutants lacking large segments of endocardium (data not shown).” It would be important to show this data.

RESPONSE:
Given that this observation is peripheral to the main theme of the manuscript and therefore distracting from the conclusions, we have now removed this statement.

Minor concerns:
1. Lines 85-89: The authors state that “In agreement with this model, the zebrafish cloche mutant lacks all endocardium, yet retains some endothelium, suggesting that the endocardium and vascular endothelium have distinct developmental origins (Stainier et al.,1995).”

However, since cloche/npas4l is not specific to the endocardium but also deletes much of the vasculature, it rather appears that endocardium and some endothelial lineages share a common origin as defined by the activity of Npas4l.

RESPONSE:
We have altered this statement to remove suggestion that *npas4l* is specific to the endocardium.
2. Line 51: Expression of nfatc1 is not endocardium-specific. RESPONSE:
We have altered the text to remove suggestion that *nfatc1* is specific to the endocardium.

3. The representation of Fig. 7B does not seem to be appropriate. The listing of all conditions, including controls, in this figure would help the reader to better perceive the defects that occur upon BMP-LOF or not and if those are rescued. This means, images and quantifications of data should be shown from the following conditions in one panel: wildtype + etv2mRNA, wildtype + control (or no) mRNA, Tg(hsp70l:nog3) + etv2mRNA, Tg(hsp70l:nog3) + control (or no) mRNA. Accordingly, this should be shown for npas4l- mRNA injection experiments.

© 2022. Published by The Company of Biologists under the terms of the Creative Commons Attribution License (https://creativecommons.org/licenses/by/4.0/).

RESPONSE:
We agree and have repeated these experiments with all conditions represented in the same figure. We have also performed the experiment for *npas4l* overexpression. This is now included in Fig 7.

4. It would be interesting, if the authors could comment and maybe show potential BMP- pathway elements that fulfil this described role of BMP signalling. Does the endocardial transcriptome data reveal any candidates?

RESPONSE:
Panther analysis of the transcriptome data shows that 3.7% of signalling pathways genes differentially regulated between fli1+ve and endocard+ tissue are components of the TGF/BMP signalling pathway. However, we show with pSMAD staining (Fig 5) that differences in the Bmp pathway between these two tissues is occurring post-transcriptionally. It, therefore, may make more sense to examine Bmp target genes or perform ChIPseq for Smad1/5/8 to identify these differences. Unfortunately, this is a much larger extension of the work and beyond the scope of the current manuscript.

5. Line 306. Here the authors mention Fig. 10 which is not present in the manuscript.

RESPONSE:
This has been amended

6. Could the authors comment on the reason for FACS sorting only Gt(endocard:egfp) high expressing cells for the RNA-seq analyses? Was this done to separate endocardium versus myocardium? Does the GO term analysis which highlights myocardial functional terms point at the fact that this marker transgene is not specific to the endocardium?

RESPONSE:
This is correct. We have now added text to page 16 of the manuscript describing this.

7.Line 359: What is meant with apical regulators?

RESPONSE:
We refer to an upstream regulator. We have now altered the text so that any confusion is avoided.

8. Line 328-330. Here, the authors claim that “These results show that the endocardium is specified earlier than previously appreciated in zebrafish development...” However, this manuscript does not provide any functional proof that cells with an expression of Gt(endocard:egfp) are indeed specified already as endocardial cells at these early stages. This paper at best suggests that the expression domain of this transgenic marker more or less fits what would be expected of a fate map for endocardium at these somitogenesis stages.

RESPONSE:
This is a reasonable comment, and we agree that we have not shown this. We have amended the text to soften the language around this claim.

Second decision letter

MS ID#: DEVELOP/2020/190421

MS TITLE: Endocardial identity is established during early somitogenesis by Bmp signalling acting upstream of npas4l and etv2/etsrp

AUTHORS: Kelly Smith, Samuel Capon, Veronica Uribe Sokolov, Nicole Dominado, and Ophelia Ehrlich

© 2022. Published by The Company of Biologists under the terms of the Creative Commons Attribution License (https://creativecommons.org/licenses/by/4.0/).

I have now received all the referees reports on the above manuscript, and have reached a decision. The referees' comments are appended below, or you can access them online: please go to BenchPress and click on the 'Manuscripts with Decisions' queue in the Author Area.

The overall evaluation is positive and we would like to publish a revised manuscript in Development, provided that the referees' comments can be satisfactorily addressed. Please attend to all of the reviewers' comments in your revised manuscript and detail them in your point-by-point response. If you do not agree with any of their criticisms or suggestions explain clearly why this is so.

We are aware that you may currently be unable to access the lab to undertake experimental revisions. If it would be helpful, we encourage you to contact us to discuss your revision in greater detail. Please send us a point-by-point response indicating where you are able to address concerns raised (either experimentally or by changes to the text) and where you will not be able to do so within the normal timeframe of a revision. We will then provide further guidance. Please also note that we are happy to extend revision timeframes as necessary.

Reviewer 1

Advance summary and potential significance to field

The manuscript by Capon and Smith describes identification of a zebrafish endocardial specific gene trap line which is used to study early stages of endocardial specification and differentiation. The authors use this line to identify the transcriptome of endocardial cells and show that these cells are transcriptionally different from vascular endothelial cells at the 8-somite stage, which is much earlier than previously demonstrated. The authors further provide data arguing for BMP and etv2 role in early endocardial specifiction and argue that etv2 functions downstream of BMP signaling in this process. Overall, the images are of high quality, results are clearly presented and provide a novel insight into the mechanism of endocardial specification.

The revised manuscript has been greatly improved and the authors have addressed most of my previous concerns. There are a few additional points that are still remaining.

Comments for the author

1. Reduction in npas4l expression in Tg(hsp:noggin3) embryos shown in Fig. S10 is not entirely convincing.
There is a significant variation in the expression level in the control embryos. It is also unclear how many embryos were analyzed total, how many showed the reduction and how many times the experiment was repeated. qPCR would provide the most accurate (and possibly easiest) estimate of the expression level although area measurements (such as done in other figures) are acceptable. Also please note how many embryos were analyzed for endocardial etv2 expression in Fig. S10 (is it 10 embryos that are shown in the figure?), how many times was experiment replicated and how many of them show reduction in endocardial etv2 expression.
2. Very low number of embryos have been analyzed in some experiments. In particular, in Fig. 7 several groups of embryos have n=4 or 5. It is unclear if these experiments have been replicated. If indeed, as listed in the methods, two replicates were performed, then this would result in only 2-3 embryos analyzed in each experiment.
3. It is intriguing that npas4l or etv2 overexpression does not result in expanded endocardial:egfp in wild-type embryos (Fig. 7C,E). However, the authors did not include a positive control which was previously suggested to demonstrate that npas4l or etv2 mRNA used in these experiments shows previously reported activity and expands expression of other vascular markers (using the same or higher doses).
4. The linkage between Tg(kdrl:eGFP) and etv2 is indeed surprising. Analysis of etv2 mutants in kdrl:GFP line has been previously reported (Casie Chetty and Sumanas, 2020, Dev Biol 465, 11-22), although it is unclear if the same kdrl:GFP allele was used in the current study. However, if etv2 and kdrl:GFP are linked then Fig. S7F is not informative because vascular patterning in etv2 uq13ks mutants cannot be assessed. A simple in situ staining for any vascular marker expression or using a different reporter line would address the question whether etv2 uq13ks mutant line recapitulates previously reported defects.

© 2022. Published by The Company of Biologists under the terms of the Creative Commons Attribution License (https://creativecommons.org/licenses/by/4.0/).

5. The text on page 6 says, 'Genes related to red blood cell development, gata1a, hbbe3 and hbae5 were enriched in the endothelial population…..' As noted previously, fli1a:GFP transgenic line labels both endothelial and blood cells. Therefore it is inaccurate to state that gata1a etc, were enriched in endothelial cells; they are most likely enriched in blood cells.

Reviewer 2

Advance summary and potential significance to field

The revised manuscript by Capon et al. is a most interesting paper related to the role of BMP signaling in endocardial progenitor cell biology. The figures are mostly of excellent quality. Most of the previous comments have been addressed satisfactorily by the authors. However, there is one main comment that still needs to be resolved. Otherwise the manuscript looks good for acceptance as is.

Comments for the author

Major comment
The authors have addressed almost all the reviewer's questions satisfactorily. Yet, whether BMP is indeed upstream of npas4l and etv2 has not been resolved in the current version of the manuscript. Although BMP inhibition reduces npas4l expression, this effect may not necessarily be direct. Alternatively, BMP and Npas4l may be acting in parallel and converge to regulate Etv2 expression. This question has also been raised by reviewer 1 (Point 4a) and by reviewer 2 (point 5). The authors have not tested the effects of a GOF activation of BMP in npas4l mutants. Also, even at 17s there is no phenotypic rescue of BMP inhibition by etv2 mRNA injection (Fig S11). Hence, the emphasis that BMP is upstream of npas4l and etv2 should be toned down throughout the paper.

Second revision

Author response to reviewers' comments

We are pleased the reviewers are supportive of our revised work, with only minor criticisms remaining. We address the remaining minor items below.

Reviewer 1 Advance Summary and Potential Significance to Field...

The manuscript by Capon and Smith describes identification of a zebrafish endocardial specific gene trap line which is used to study early stages of endocardial specification and differentiation. The authors use this line to identify the transcriptome of endocardial cells and show that these cells are transcriptionally different from vascular endothelial cells at the 8-somite stage, which is much earlier than previously demonstrated. The authors further provide data arguing for BMP and etv2 role in early endocardial specifiction and argue that etv2 functions downstream of BMP signaling in this process. Overall, the images are of high quality, results are clearly presented and provide a novel insight into the mechanism of endocardial specification.

The revised manuscript has been greatly improved and the authors have addressed most of my previous concerns. There are a few additional points that are still remaining.

Reviewer 1 Comments for the Author...

1. Reduction in npas4l expression in Tg(hsp:noggin3) embryos shown in Fig. S10 is not entirely convincing. There is a significant variation in the expression level in the control embryos.

This is correct. It reflects the variation that exists in most biological contexts however we disagree and consider that it is clear that npas4l expression is reduced in Tg(hsp:noggin3) embryos compared with transgenic negative controls.

© 2022. Published by The Company of Biologists under the terms of the Creative Commons Attribution License (https://creativecommons.org/licenses/by/4.0/).

It is also unclear how many embryos were analyzed total, how many showed the reduction and how many times the experiment was repeated.

As described in the methods, all experiments were performed twice. Figure S10 represents one of the two experiments. We have now added additional information in the Figure legend to make this clear.

qPCR would provide the most accurate (and possibly easiest) estimate of the expression level, although area measurements (such as done in other figures) are acceptable. Also please note how many embryos were analyzed for endocardial etv2 expression in Fig.
S10 (is it 10 embryos that are shown in the figure?), how many times was experiment replicated and how many of them show reduction in endocardial etv2 expression.

Experimental and biological replicate numbers have now been added to the Figure legend.

2. Very low number of embryos have been analyzed in some experiments. In particular, in Fig. 7 several groups of embryos have n=4 or 5. It is unclear if these experiments have been replicated. If indeed, as listed in the methods, two replicates were performed, then this would result in only 2-3 embryos analyzed in each experiment.

This is correct and we believe sufficient because of the nature of the experiment and robustness of the result. Fig 7 reports a rescue experiment: No transcript expression was observed in the control (endocard:GFP expression following hs:noggin, n = 9) and a clear and robust rescue was observed for the treatment (endocard:GFP expression following hs:noggin + npas4l injection; n = 4). Biological replicates of n=4 is sufficient for a clear result, providing a definitive conclusion.

3. It is intriguing that npas4l or etv2 overexpression does not result in expanded endocardial:egfp in wild-type embryos (Fig. 7C,E). However, the authors did not include a positive control which was previously suggested to demonstrate that npas4l or etv2 mRNA used in these experiments shows previously reported activity and expands expression of other vascular markers (using the same or higher doses).

We agree, it is intriguing and suggests the endocardial spatial domain is tightly regulated. For example, when we overexpress npas4l or etv2 on a hs:noggin background, we rescue endocard:gfp expression. Whilst the rescued expression domain is dysmorphic compared with wildtype (Fig 7C & E), it is still restricted to the same region (bilateral expression in the ALPM). This suggests that this is the only area competent to respond to npas4l or etv2 to express endocard:gfp.

4. The linkage between Tg(kdrl:eGFP) and etv2 is indeed surprising. Analysis of etv2 mutants in kdrl:GFP line has been previously reported (Casie Chetty and Sumanas, 2020, Dev Biol 465, 11-22), although it is unclear if the same kdrl:GFP allele was used in the current study. However, if etv2 and kdrl:GFP are linked, then Fig. S7F is not informative because vascular patterning in etv2 uq13ks mutants cannot be assessed. A simple in situ staining for any vascular marker expression or using a different reporter line would address the question whether etv2 uq13ks mutant line recapitulates previously reported defects.

Whilst we believe the phenotypes shown here are consistent with previously published etv2 alleles, including cardiac oedema and a failure to remodel the caudal vein plexus (a vascular patterning phenotype), we can appreciate the reviewers hesitancy. To address this, we have performed imaging of the $etv2^{uq13ks}$ allele on the Gt(endocard:egfp) background and include it in the resubmission. The Gt(endocard:egfp) line has venous endothelial expression by 48 hpf (as previously reported by Bussmann et al., Development, 2010). We have utilised this to investigate intersegmental vessel (ISV) sprouting in our etv2 allele. We observe both a loss of ISV sprouting and ectopic expression in skeletal muscle fibres (revised Figure S7, inset). Both phenotypes are consistent with previously published data on other etv2 alleles (Chestnut et al., 2020).

With regards to linkage, we agree it is very surprising. Our Tg(kdrl:eGFP) allele originates from the Hubrecht laboratories, which sourced the original s843 allele from the Stainier laboratory, where it was created (Jin et al., 2005). This is the same allele reported by Casie Chetty and Sumanas, 2020.

© 2022. Published by The Company of Biologists under the terms of the Creative Commons Attribution License (https://creativecommons.org/licenses/by/4.0/).

What is unclear is how tightly linked these loci are and whether Casie Chetty and Sumanas maintained their etv2 allele on the Tg(kdrl:eGFP)^{s843} background. For example, we have previously mapped the gene Tmem2 and found it linked to the Tg(fli1a:egfp)^{y1} locus however, we get rare recombinants and can therefore maintain the Tmem2 allele with ease on the Tg(fli1a:egfp)^{y1} background, once a recombinant is identified and used to propagate the line. It is not stated if this is the case in Casie Chetty and Sumanas, 2020. What we are clear about is that we have observed linkage between the etv2 locus and the kdrl:eGFP transgene for the s843 allele. Given that no reports of its genomic location exist, we considered it appropriate to include so as not to waste this information.

5. The text on page 6 says, 'Genes related to red blood cell development, gata1a, hbbe3 and hbae5 were enriched in the endothelial population…..' As noted previously, fli1a:GFP transgenic line labels both endothelial and blood cells. Therefore it is inaccurate to state that gata1a etc, were enriched in endothelial cells; they are most likely enriched in blood cells.

This is well noted, and we have now corrected it.

Reviewer 2 Advance Summary and Potential Significance to Field...

The revised manuscript by Capon et al. is a most interesting paper related to the role of BMP signaling in endocardial progenitor cell biology. The figures are mostly of excellent quality. Most of the previous comments have been addressed satisfactorily by the authors. However, there is one main comment that still needs to be resolved. Otherwise the manuscript looks good for acceptance as is.

Reviewer 2 Comments for the Author...

Major comment

The authors have addressed almost all the reviewer's questions satisfactorily. Yet, whether BMP is indeed upstream of npas4l and etv2 has not been resolved in the current version of the manuscript. Although BMP inhibition reduces npas4l expression, this effect may not necessarily be direct.

We agree and have not claimed that it is direct, merely upstream. We show data that demonstrates in numerous ways, and with consistency, that Bmp signalling acts upstream of npas4l and etv2, and that these act upstream of endocardial differentiation.

Alternatively, BMP and Npas4l may be acting in parallel and converge to regulate Etv2 expression.

We have shown here that npas4l expression is reduced upon Bmp inhibition. Whilst we cannot rule out that Bmp and npas4l may also function in parallel to converge on etv2, we do know definitively that npas4l expression is reduced upon Bmp inhibition and that npas4l has previously been shown to act directly upstream of etv2 by binding to its promoter (Marass et al., Development, 2019).

This question has also been raised by reviewer 1 (Point 4a) and by reviewer 2 (point 5). The authors have not tested the effects of a GOF activation of BMP in npas4l mutants.
Also, even at 17s there is no phenotypic rescue of BMP inhibition by etv2 mRNA injection (Fig S11). Hence, the emphasis that BMP is upstream of npas4l and etv2 should be toned down throughout the paper.

Whilst we have not shown the GOF effect of BMP in npas4l mutants, we have shown it for etv2 as well as the reciprocal: that npas4l or etv2 overexpression is epistatic to BMP inhibition. This is sufficient evidence to demonstrate that Bmp is acting upstream of npas4l and etv2.

© 2022. Published by The Company of Biologists under the terms of the Creative Commons Attribution License (https://creativecommons.org/licenses/by/4.0/).

Third decision letter

MS ID#: DEVELOP/2020/190421

MS TITLE: Endocardial identity is established during early somitogenesis by Bmp signalling acting upstream of npas4l and etv2/etsrp

AUTHORS: Kelly Smith, Samuel Capon, Veronica Uribe Sokolov, Nicole Dominado, and Ophelia Ehrlich
ARTICLE TYPE: Research Article

I am happy to tell you that your manuscript has been accepted for publication in Development, pending our standard ethics checks.

© 2022. Published by The Company of Biologists under the terms of the Creative Commons Attribution License (https://creativecommons.org/licenses/by/4.0/).
